# A type III polyketide synthase cluster in the phylum *Planctomycetota* is involved in alkylresorcinol biosynthesis

**DOI:** 10.1007/s00253-024-13065-x

**Published:** 2024-02-26

**Authors:** Lars Milke, Moses Kabuu, Renè Zschoche, Jochem Gätgens, Karin Krumbach, Kim-Loreen Carlstedt, Carmen E. Wurzbacher, Sven Balluff, Christine Beemelmanns, Christian Jogler, Jan Marienhagen, Nicolai Kallscheuer

**Affiliations:** 1 Institute of Bio- and Geosciences, IBG-1: Biotechnology, Forschungszentrum Jülich, 52425 Jülich, Germany; 2 Department of Microbial Interactions, Institute for Microbiology, Friedrich Schiller University, 07743 Jena, Germany; 3 Helmholtz Institute for Pharmaceutical Research Saarland (HIPS), 66123 Saarbrücken, Germany; 4 Saarland University, Saarbrücken, Germany; 5 Institute of Biotechnology, RWTH Aachen University, Worringer Weg 3, 52074 Aachen, Germany

**Keywords:** Planctomycetes, *Corynebacterium glutamicum*, Polyketide-derived compounds, Hierridin, Chemical mediators

## Abstract

**Abstract:**

Members of the bacterial phylum *Planctomycetota* have recently emerged as promising and for the most part untapped sources of novel bioactive compounds. The characterization of more than 100 novel species in the last decade stimulated recent bioprospection studies that start to unveil the chemical repertoire of the phylum. In this study, we performed systematic bioinformatic analyses based on the genomes of all 131 described members of the current phylum focusing on the identification of type III polyketide synthase (PKS) genes. Type III PKSs are versatile enzymes involved in the biosynthesis of a wide array of structurally diverse natural products with potent biological activities. We identified 96 putative type III PKS genes of which 58 are encoded in an operon with genes encoding a putative oxidoreductase and a methyltransferase. Sequence similarities on protein level and the genetic organization of the operon point towards a functional link to the structurally related hierridins recently discovered in picocyanobacteria. The heterologous expression of planctomycetal type III PKS genes from strains belonging to different families in an engineered *Corynebacterium glutamicum* strain led to the biosynthesis of pentadecyl- and heptadecylresorcinols. Phenotypic assays performed with the heterologous producer strains and a constructed type III PKS gene deletion mutant suggest that the natural function of the identified compounds differs from that confirmed in other bacterial alkylresorcinol producers.

**Key points:**

*• Planctomycetal type III polyketide synthases synthesize long-chain alkylresorcinols.*

*• Phylogenetic analyses suggest an ecological link to picocyanobacterial hierridins.*

*• Engineered C. glutamicum is suitable for an expression of planctomycete-derived genes.*

**Supplementary Information:**

The online version contains supplementary material available at 10.1007/s00253-024-13065-x.

## Introduction

Members of the phylum *Planctomycetota* have gained attention due to their intriguing cellular characteristics. From early research on the phylum starting in 1924 until 2010, only ten species could be brought into axenic culture and have been validly described (Ivanova and Dedysh 2012). Based on intensive worldwide sampling and strain isolation campaigns conducted in the last decade combined with the development of more selective cultivation methods, about 100 novel species have been added to the open strain collection of characterized planctomycetes (Lage and Bondoso 2012; Wiegand et al. 2020, 2018).

Especially *Planctomycetia*, the class with the currently highest number of described species within the phylum, exhibits biological features different from other members of this phylum (Vitorino and Lage 2022). Dimorphic lifestyles, large genomes of up to 12.4 Mb, high numbers of hypothetical protein-encoding genes, and predicted natural product biosynthetic gene clusters (BGCs) have been described in members of this class (Kallscheuer and Jogler 2021; Kulichevskaya et al. 2017; Overmann et al. 2017). Whereas members of the other classes propagate by binary fission (Wiegand et al. 2020), *Planctomycetia* members propagate by an uncommon form of asymmetric cell division (often referred to as “budding”).

Due to molecular and genomic insights gained over the last decade, research in the entire phylum is currently in a transition phase from the pure description of novel isolates to an application-based exploration of the biotechnological potential of its members. This is reflected in the recent discovery of the first natural products formed by members of the phylum (Kallscheuer et al. 2020, 2019b; Panter et al. 2019; Sandargo et al. 2020; Santana-Molina et al. 2022). Hitherto, all natural compounds produced by planctomycetes were discovered by untargeted chromatographic analyses. BGCs involved in the production of these empirically confirmed compounds escaped an in silico prediction with bioinformatic tools such as antiSMASH (Blin et al. 2021). In contrast, BGCs belonging to well-characterized compound classes, for example, polyketides and non-ribosomal peptides, can be reliably detected also in planctomycetal genomes. Still, the bioinformatical prediction algorithms are trained with BGCs from well-explored bacterial phyla, for example, actinobacteria or cyanobacteria. This might explain why most of the planctomycetal BGCs do not cluster with BGCs of the MIBiG (Minimum Information about a Biosynthetic Gene cluster) database (Wiegand et al. 2020). This supports the role of planctomycetes as yet for the most part untapped sources of natural products with potential antimicrobial or health-promoting activities (Kallscheuer et al. 2020).

An important class of enzymes involved in natural product biosynthesis are polyketide synthases (PKSs). PKSs use an acyl carrier protein- (ACP) or coenzyme A- (CoA) activated starter unit and catalyze multiple rounds of chain elongation (polyketide formation) using malonyl-CoA as extension unit. Known PKSs are subdivided into three distinct subclasses (types I, II, and III) that reflect different levels of complexity regarding protein domain architectures and catalyzed reaction cascades (Nivina et al. 2019).

In this study, we explore the function of planctomycetal type III PKSs. Enzymes of this class are involved in the biosynthesis of a wide range of bioactive compounds among all domains of life. The best-characterized type III PKSs are plant stilbene and chalcone synthases that catalyze the first committed step of polyphenol biosynthesis pathways (Andrés-Lacueva et al. 2009). Polyphenols, such as stilbenes and flavonoids, show potent health-promoting activities in humans (Pandith et al. 2019; Rozmer and Perjési 2016). Type III PKSs are relatively small enzymes with a length of 250–450 amino acids. They harbor a single active site that typically performs 3–10 rounds of malonyl-CoA-dependent chain elongation and finally cyclize and aromatize the polyketide precursor to a di- or trihydroxylated aromatic ring. In contrast to the other two classes, type III PKSs do not require posttranslational activation by transfer of a phosphopantetheinyl residue to a carrier domain. This facilitates the functional expression of type III PKS-encoding genes in various heterologous hosts. However, the strict regulation of malonyl-CoA biosynthesis and maintenance of only low levels for fatty acid biosynthesis in bacteria turned out to be a major bottleneck for the engineered biosynthesis of polyketide-derived polyphenolic compounds in microorganisms (Lim et al. 2011; Milke et al. 2018; Milke and Marienhagen 2020).

For testing the expression of yet uncharacterized planctomycete-derived type III PKS genes, we employed a previously engineered *Corynebacterium glutamicum* strain (Milke et al. 2020). Engineered strains of this Gram-positive bacterium are typically used for the production of amino acids (Eggeling and Bott 2015), but in the recent years, various (aromatic) natural compound-producing strains have been constructed (Kogure and Inui 2018; Milke et al. 2020; Wolf et al. 2021).

## Materials and methods

### Analysis of planctomycetal genomes and construction of phylogenetic trees

The reference genomes of all characterized species of the phylum *Planctomycetota* were retrieved from GenBank (Table S1). The prediction of BGCs potentially involved in natural product biosynthesis was performed with antiSMASH version 7.0.0 (Blin et al. 2021). The analysis was performed with relaxed strictness and all extra features activated.

For tree construction, the 16S rRNA gene sequences obtained from the planctomycetal genomes were aligned with ClustalW (Thompson et al. 2003). A maximum likelihood phylogenetic tree was calculated from the alignment with FastTree 2.1 (Price et al. 2010) employing the GTR + CAT model and 1000 bootstrap replications. Three 16S rRNA genes of bacterial strains from the *Planctomycetota-Verrucomicrobiota-Chlamydiota* (PVC) superphylum outside of the phylum *Planctomycetota*, namely *Opitutus terrae* (NCBI acc. no. AJ229235), *Kiritimatiella glycovorans* (acc. no. NR_146840), and *Lentisphaera araneosa* (acc. no. NR_027571), were used as outgroup.

The locus tags of all planctomycetal type III PKS genes were retrieved from the antiSMASH results, and the encoded protein sequences were extracted from the annotated genomes. Sequences of plant and bacterial type III PKS proteins with known product specificity were obtained from UniProt (or NCBI when the protein was not included in the UniProt database). Alignment and tree construction were performed as described above, but the JTT + CAT model was employed for protein sequences. Phylogenetic trees were visualized with iTOL v6 (Letunic and Bork 2021).

### Medium preparation and cultivation

*C. glutamicum* was routinely cultivated aerobically at 30 °C in brain heart infusion (BHI) medium (Difco Laboratories, Detroit, USA) or defined CGXII medium with 4% (w/v) glucose as sole carbon and energy source (Keilhauer et al. 1993). *E. coli* DH5α was used for plasmid construction and was cultivated in LB medium (Bertani 1951) at 37 °C. Where appropriate, kanamycin (50 mg/L for *E. coli* or 15 mg/L for *C. glutamicum*) and/or spectinomycin (100 mg/L for *E. coli* and *C. glutamicum*) was added to the medium. Bacterial growth was followed by measuring the optical density at 600 nm (OD_600_). *C. glutamicum* was grown for 6–8 h in test tubes with 5 mL BHI medium on a rotary shaker at 170 rpm (first preculture) and was subsequently inoculated into 50 mL defined CGXII medium with 4% (w/v) glucose in 500 mL baffled Erlenmeyer flasks (second preculture). The cell suspensions were cultivated overnight on a rotary shaker at 130 rpm. The main culture was inoculated to an OD_600_ of 5.0 in defined CGXII medium with 4% (w/v) glucose. The heterologous episomal gene expression was induced 2 h after inoculation by the addition of 1 mM isopropyl β-D-1-thiogalactopyranoside (IPTG).

### Construction of plasmids and strains

All bacterial strains and plasmids used in this study as well as their relevant characteristics are listed in Table 1. Standard protocols of molecular cloning, such as PCR, DNA restriction, and ligation (Sambrook and Russell 2001), were carried out for recombinant DNA work. Techniques specific for *C. glutamicum*, e.g., electroporation for transformation of strains, were done as described previously (Eggeling and Bott 2005). All enzymes were obtained from ThermoScientific (Schwerte, Germany). Codon-optimized synthetic genes for *C. glutamicum* ATCC 13032 were obtained from Integrated DNA Technologies (IDT, Leuven, Belgium). Genes were PCR-amplified with the KOD polymerase Master mix (Merck Millipore, Darmstadt, Germany) using primers that introduce flanking homology regions of the acceptor plasmids pMKEx2 or pEKEx3 (Table 2). The PCR products were subsequently used for subcloning into pMKEx2 linearized with *Bam*HI and *Nco*I using Gibson Assembly. The assembly reaction was incubated for 60 min at 50 °C. In the same manner, PCR products were subcloned into the *Bam*HI/*Eco*RI-linearized plasmid pEKEx3. All constructed plasmids were finally verified by check-PCR using primers that bind outside of the inserted sequences and by DNA sequencing at Eurofins Genomics (Ebersberg, Germany). The check-PCR was performed with the DreamTaq Green Mastermix (ThermoFisher) with the following program: initial denaturation 5 min, 95 °C; 33 cycles of denaturation (95 °C, 30 s), annealing (57 °C, 20 s), and elongation (72 °C, 1 min/kb), and a final elongation step (95 °C, 5 min).

**Table 1:** Strains and plasmids used in this study

Strain or plasmid	Relevant characteristics	Source or reference
**Strains**		
*E. coli* DH5α	F– Φ80*lacZ*ΔM15 Δ(*lacZYA-argF*)U169 *recA1 endA1 hsdR17* (rK–, mK +) *phoA supE44* λ– *thi-1 gyrA96 relA1*	Invitrogen (Karlsruhe, Germany)
*E. coli* Δ*tolC*	DSM 104619, harbors an IS5 transposon insertion in the open reading frame of *tolC*	DSMZ (Braunschweig,Germany)
*B. subtilis*	type strain DSM 10^ T^	DSMZ (Braunschweig,Germany)
*C. glutamicum* M-CoA	*C. glutamicum* MB001(DE3) derivative with in-frame deletions of cg0344-47, cg0503, cg2625-40 and cg1226 involved in the degradation of aromatic compounds; harboring a chromosomally encoded codon-optimized *4 cl* gene coding for a 4-coumarate: CoA ligase from *Petroselinum crispum* under control of the T7 promoter; replacement of the native citrate synthase gene (*gltA*) promoter with the *dapA* promoter variant C5, mutated fasO binding sites upstream of the acetyl-CoA carboxylase-encoding genes *accBC* and *accD1*, two nucleotide exchanges in the promoter region of the hexose transporter-encoding gene *iolT1* and in-frame deletion of the pyruvate carboxylase gene *pyc*	(Milke et al. 2019)
*S. maiorica* ΔMal15_23020::*cat*	derivative of the type strain Mal15^T^ in which the open reading frame of the type III PKS gene Mal15_23020 is replaced by the *cat* gene under control of a constitutive promoter	This study
*S. maiorica* ΔMal15_51000::*cat*	derivative of the type strain Mal15^T^ in which the open reading frame of Mal15_51000 (putative serine/threonine protein kinase) is replaced by the *cat* gene under control of a constitutive promoter	This study
**Plasmids**		
pCJ003x(cat)	*cm*^r^, *E. coli* vector (pUC ori_*Ec*_)	This study
pMKEx2	*kan*^r^; *E. coli-C.* *glutamicum* shuttle vector (*lacI*, P_T7_, lacO1, pHM1519 ori_*Cg*_; pACYC177 ori_*Ec*_)	(Kortmann et al. 2015)
pMKEx2_t3pks_*AcCg*_	pMKEx2 derivate for the expression of the type III polyketide synthase gene from *Alienimonas californiensis* CA12^T^ (locus tag CA12_06310, codon-optimized for *C. glutamicum*)	This study
pMKEx2_t3pks_*GmCg*_	pMKEx2 derivate for the expression of the type III polyketide synthase gene from *Gimesia maris* DSM 8797^ T^ (locus tag PM8797T_03239, codon-optimized for *C. glutamicum*)	This study
pMKEx2_t3pks_*PlCg*_	pMKEx2 derivate for the expression of the type III polyketide synthase gene from *Planctopirus limnophila* DSM 3776^ T^ (locus tag Plim_0466, codon-optimized for *C. glutamicum*)	This study
pMKEx2_t3pks_*RbCg*_	pMKEx2 derivate for the expression of the type III polyketide synthase gene from *Rhodopirellula baltica* SH1^T^ (locus tag RB8853, codon-optimized for *C. glutamicum*)	This study
pMKEx2_t3pks_*SfCg*_	pMKEx2 derivate for the expression of the type III polyketide synthase gene from *Saltatorellus ferox* Poly30^T^ (locus tag Poly30_13920, codon-optimized for *C. glutamicum*)	This study
pMKEx2_t3pks_*SmCg*_	pMKEx2 derivate for the expression of the type III polyketide synthase gene from *Stieleria maiorica* Mal15^T^ (locus tag Mal15_23020, codon-optimized for *C. glutamicum*)	This study
pEKEx3	*spec*^r^; *E. coli-C.* *glutamicum* shuttle vector (*lacI*, P_*tac*_, lacO1, pBL1ori_*Cg*_; pUCori_*Ec*_)	(Gande et al. 2007)
pEKEx3_mtr_*GmCg*_-fdo_*GmCg*_	pEKEx3 derivate for the expression of the genes coding for a putative methyltransferase (*mtr,* locus tag PM8797T_03249) and FAD-dependent oxidoreductase (*fdo, locus tag* PM8797T_03244) from *Gimesia maris* DSM 8797^ T^ (codon-optimized for *C. glutamicum*)	This study
pEKEx3_mtr1_*SfCg*_-mtr2_*SfCg*_	pEKEx3 derivate for the expression of the genes coding for two putative methyltransferases (*mtr1,* locus tag Poly30_13900) and FAD-dependent oxidoreductase (*mtr2,* locus tag Poly30_13910) from “*Saltatorellus ferox*” Poly30^T^ (codon-optimized for *C. glutamicum*)	This study

*cm*^r^ chloramphenicol resistance, *kan*^r^ kanamycin resistance, *spec*^r^ spectinomycin resistance

**Table 2:** Oligonucleotides used in this study

Oligonucleotide	Sequence (5′-3′)
check-pMKEx2-s	CCGATCTTCCCCATCGGTGATGTCGGCGATATAG
check-pMKEx2-s	ACCTGCAGCTTAATGCGCCGCTACAGG
check-pEKEx3-s	GTCGTAAATCACTGCATAATTCGTGTCGCTCAAGG
check-pEKEx3-as	CAGGCTGAAAATCTTCTCTCATCCGCCAAAACAG
t3pksAc-s	ACTTTAAGAAGGAGATATACCATGGTAAGGAGGACAGCTATGTCGGGTATTGGAACTG
t3pksAc-as	GGCCGCGTCGACTTGTACAGGATCCAGGACTAGTTTCCAGAGTACTATTAACCAAACAGGGCAGC
t3pksGm-s	ACTTTAAGAAGGAGATATACCATGGTAAGGAGGACAGCTATGAGCTTCGAAATTTTGG
t3pksGm-as	GGCCGCGTCGACTTGTACAGGATCCAGGACTAGTTTCCAGAGTACTATTATTTGACAAGAGCAGC
t3pksPl-s	ACTTTAAGAAGGAGATATACCATGGTAAGGAGGACAGCTATGGCAATGCTGATCTCAG
t3pksPl-as	GGCCGCGTCGACTTGTACAGGATCCAGGACTAGTTTCCAGAGTACTATTAGATGTTCTCGGCTTTC
t3pksRb-s	ACTTTAAGAAGGAGATATACCATGGTAAGGAGGACAGCTATGACCGCTCAGATTCTG
t3pksRb-as	GGCCGCGTCGACTTGTACAGGATCCAGGACTAGTTTCCAGAGTACTATTAGCGGACAACTGCAAC
t3pksSf-s	ACTTTAAGAAGGAGATATACCATGGTAAGGAGGACAGCTATGCGTATCGCTTCCGTC
t3pksSf-as	GGCCGCGTCGACTTGTACAGGATCCAGGACTAGTTTCCAGAGTACTATTAGCACCACTCAAACAGAAG
t3pksSm-s	ACTTTAAGAAGGAGATATACCATGGTAAGGAGGACAGCTATGATCATCGACGGTTTG
t3pksSm-as	GGCCGCGTCGACTTGTACAGGATCCAGGACTAGTTTCCAGAGTACTATTATCCAATCAATGCGGC
mtrGm-s	CCTGCAGGTCGACTCTAGAGGATCCAAGGAGGTCATATCATGAATTTCAAAATCCGTCAG
mtrGm-as	ACGATTCCTCCTTGTACATTCACGGGAGCTCTTATTTACGGCTCCATTTC
fdoGm-s	GAGCTCCCGTGAATGTACAAGGAGGAATCGTATGACTTTGACGTCTTCAC
fdoGm-as	CTGTAAAACGACGGCCAGTGAATTCTTAATGGTTAAGCCGTGC
mtr1Sf-s	CCTGCAGGTCGACTCTAGAGGATCCAAGGAGGTCATATCATGGCTCAAGCACAGAATG
mtr1Sf-as	ACGATTCCTCCTTGTACATTCACGGGAGCTCTTATGCCTGTGCCTTCTC
mtr2Sf-s	GAGCTCCCGTGAATGTACAAGGAGGAATCGTATGACGGAGATTGCCCTTCGCC
mtr2Sf-as	CTGTAAAACGACGGCCAGTGAATTCTTACGGCCGCTGGGGCGT
XhoI-Mal15_23020-up-s	CGTCTCGAGGACGGTCTCCAACAAGCTCTCGGCAAGTTG
KpnI-Mal15_23020-up-as	TCTGGTACCCGTGCCCAGGCCGTCAATGATCATCAC
XbaI-Mal15_23020-down-s	CGTTCTAGAATCGAAGCGGCGTTAATCGGTTAGCGGTTG
HindIII-Mal15_23020-down-as	TCTAAGCTTATCCCCGAATTGGTCCAAGAATTGAAACTCAGCAG
check-Mal15_23020-s	GATAACGTGTGTTGGTGGTCAGTTGAACGATCC
check-Mal15_23020-as	CGTCCGGCGGCAATGATCCTGATGACATC
XhoI-Mal15_51000-up-s	CGTCTCGAGCCTGGAAGACGCCGATGCATCCCCAC
KpnI-Mal15_51000-up-as	TCTGGTACCCCGTTTGGTTTTGACAGTCGTCATCTTCCGTTG
XbaI-Mal15_51000-down-s	CGTTCTAGACGCTGCGATATCAATTGGAGCTAAGCCCAGTG
XbaI-Mal15_51000-down-as	CGTTCTAGAACCGTACTTGGTCTCGGCCTTGAGCAAAC
check-Mal15_51000-s	GCGGGTTATTGGTGAGCTAGTTTTCAGTCAACCGTCCTAC
check-Mal15_51000-as	GATCTGAAACCCCGCTTCGACACTCTCCAG

Restriction sites used for cloning are underlined

Construction of gene deletion mutants in *Stieleria maiorica.*

For the targeted gene inactivation in *S. maiorica* Mal15^T^ (= DSM 100215^ T^ = LMG 29790^ T^), the flanking regions up- and downstream of the coding sequence of a specific gene (approximately 1500 bp each) were amplified with the Q5 polymerase Master mix (New England Biolabs) using the recommended standard PCR protocol and subsequently cloned into the plasmid pCJ003x(cat). The amplified homology regions still included the first and last 24 nucleotides of the coding sequence of the inactivation target. In the final plasmid, the inserted homology arms flank the chloramphenicol resistance gene *cat* including its promoter. Prior to the transformation of *S. maiorica*, 2 µg of plasmid DNA was linearized with a single-cutting restriction enzyme that cleaves outside of the assembled insert region (upstream homology region–P_*cat*_-*cat*–downstream homology region) and purified with the NucleoSpin Gel and PCR Clean‑up Kit (Macherey–Nagel, Düren, Germany).

Electrocompetent cells of *S. maiorica* Mal15^T^ were prepared from 200 mL of an exponentially growing culture (OD_600_ of 0.5–0.7) in M3H NAG ASW medium. The medium composition was published in a previous study (Sandargo et al. 2020). The cells were harvested by centrifugation (4500 rpm, 4 °C, 20 min) and washed twice with sterile 10% (v/v) glycerol. Cells obtained after the second washing step were mixed with the linearized plasmid DNA and transferred to a Gene Pulser Electroporation Cuvette with 0.2 cm gap (Biorad). The electroporation was performed with a GenePulser Xcell (Biorad) with the following parameters: 2500 V, 25 µF, 200 Ω, pulsing time 5 ms. The cell suspension was immediately transferred to 4 mL M3H NAG ASW medium and cultivated for 2 h at 28 °C on a rotary shaker. The cells were harvested by centrifugation (4500 rpm, 20 °C, 6 min) and ultimately streaked on M3H NAG ASW plates containing 34 mg/L chloramphenicol that were incubated at 28 °C. Colonies obtained after 7–10 days were re-streaked and checked for the expected gene inactivation by PCR (DreamTaq Green Master mix).

### Two-phase liquid–liquid extraction of the culture broth and GC-TOF–MS analysis

Extracts of the fermented culture broth (medium and cells) were prepared using a two-phase extraction protocol (Kallscheuer et al. 2016). Briefly, per strain six times 1 mL of culture broth was mixed with the same volume of ethyl acetate. The mixture was shaken in an Eppendorf Thermomixer (1200 rpm, 10 min., 20 °C). After a centrifugation step (13,000 rpm, 6 min, room temperature), 800 µL of the upper ethyl acetate phase was transferred to a solvent-resistant deep-well plate and the solvent was evaporated to dryness in a fume hood overnight. On the next day, the dried extract was pooled in 150 µL acetonitrile per culture and stored at − 20 °C until further processing.

For GC-TOF–MS analysis (Paczia et al. 2012), 130 µL aliquots of the samples were lyophilized overnight in a Christ LT-105 freeze drier (Martin Christ Gefriertrocknungsanlagen). The dried samples were consecutively derivatized with 50 µL 20 mg/mL *O*-methylhydroxylamine (MeOX) (Sigma Aldrich) in pyridine (Honeywell) for 90 min at 30 °C and 600 rpm in an Eppendorf Thermomixer followed by an incubation with additional 80 µL of MSTFA (*N*-acetyl-*N*-(trimethylsilyl)-trifuoroacetamide) (Sigma-Aldrich) for 90 min at 40 °C and 600 rpm. For the determination of the derivatized metabolites, an Agilent 8890N double SSL gas chromatograph (Agilent, Waldbronn, Germany) equipped with an L-PAL3-S15 liquid autosampler was used, coupled to a LECO Pegasus GC-HRT + 4D high-resolution time of flight mass spectrometer (LECO, Mönchengladbach, Germany). The system was controlled by the LECO ChromaToF software. For 1D GC analysis, the back injector was equipped with a 30 m Agilent EZ-Guard VF-5 ms + 10 m guard column (Agilent, Waldbronn, Germany). The front injector was equipped with a 30 m HP 5-ms Ui column (HP) connected to a 2 m Rtxi17 (Restek) in a secondary oven. A constant helium flow was adjusted to 1 mL/min for the active (back) injector and column and to 0.5 mL/min for the passive (front) injector. The GC temperature program started at 60 °C with a hold time of 2 min, followed by a temperature ramp of + 12 °C/min up to the final temperature of 300 °C, hold time of 8 min. This leads to a total run time of 30 min. The secondary oven temperature offset was set to 15 °C above the first oven temperature. The transfer line temperature was set to 300 °C. The TOF–MS was operated in positive electron impact [EI]^+^ mode at an electron energy of 70 eV. The ion source temperature was set to 250 °C. The mass spectrometer was tuned and calibrated with the mass fragmentation pattern of PFTBA (perfluorotributylamine). During analysis, the accurate masses were corrected to a single point lockmass of Heptacosa as an external reference at *m/z* 218.9856. 1D Data acquisition was performed in stick mode with a rate of 12 scans/s. To annotate known metabolites, a baseline noise-corrected fragmentation pattern together with the corresponding current RI value (Retention time Index) was compared to the in-house accurate *m/z* database JuPoD and the commercial nominal *m/z* database NIST20 (National Institute of Standards and Technology, USA). Unknown peaks were identified by a virtual reconstruction of the derivatized metabolite structure via the measured baseline noise-corrected accurate mass *m/z* fragment pattern in comparison to an accurate *m/z* fragment register inside the JuPoD main library and were subsequently verified by virtual derivatization and fragmentation of the predicted structure.

### Antimicrobial activity assay

For testing a potential antimicrobial activity of the compounds produced by planctomycetal type III PKSs in *C. glutamicum*, the constructed strains were cultivated as described above. Three days after induction of the episomal gene expression, the entire culture volume (50 mL per strain) was extracted using the two-phase extraction protocol with ethyl acetate described above. The up-scaled procedure was performed in 50 mL Falcon tubes. After the centrifugation step, the organic phase was collected in a round bottom flask and the ethyl acetate was evaporated in a rotary evaporator (Rotavapor R-114 connected to a B-480 water bath, Büchi) at 40 °C and 240 mbar. Each extract was evaporated to dryness and resuspended in 200 µL methanol. Subsequently, 20 µL of the resuspended extract was added to a filter disk (6 mm) and allowed to dry for ca. 20 min. The disks were placed on LB plates inoculated with the indicator strains *E. coli* ∆*tolC* (Donner et al. 2017) and *Bacillus subtilis* DSM10^T^. The plates were incubated at 28 °C. Discs with kanamycin (10 µg per disc) were used as a positive control, while 20 µL methanol and 20 µL sterile ddH_2_O served as negative controls. The plates were inspected for zones of inhibition after 24 h of incubation.

### β-Lactam antibiotic sensitivity assays

To check for an increased resistance of the constructed type III PKS gene-expressing *C. glutamicum* strains to β-lactam antibiotics, the empty vector control strain and the eight expression strains were cultivated as described above. Three days after the induction of the episomal expression of planctomycete-derived genes, 100 µL of the culture was streaked on CGXII plates supplemented with 4% (w/v) glucose, 1 mM IPTG, 15 mg/L kanamycin (and 100 mg/L spectinomycin for the strains harboring two plasmids). Two ampicillin minimal inhibitory concentration (MIC) test stripes (0.016–256 μg/mL, Bestbion dx) were placed on each plate, and all plates were incubated for 2 days at 28 °C. The MIC values were visually determined from the obtained zones of inhibition. The constructed gene inactivation mutants of *S. maiorica* Mal15^T^ were tested for sensitivity to ampicillin using the same protocol but were streaked on M3H NAG ASW plates with 34 mg/L chloramphenicol and incubated at 28 °C for 2 days.

## Results

Distribution of type III polyketide synthase genes in the phylum *Planctomycetota.*

The type strain genomes of all currently described species of the phylum *Planctomycetota* were analyzed for the presence of type III PKS-encoding genes using the antiSMASH pipeline. The current collection of effectively or validly described members of the phylum includes 137 species (as of June 2023). The genomes of the type strains of six species have not been sequenced yet. The analysis of 131 genomes (Table S1) uncovered that 91 genomes encode at least one type III PKS. A single strain, *Thalassoroseus polymorphus* Mal48^T^, carries two type III PKS genes (Rivas-Marin et al. 2020), whereas even three type III PKS genes were found in two strains, *Lacipirellula parvula* PX69^T^ and *Lacipirellula limnantheis* I41^T^ (Dedysh et al. 2020; Kallscheuer et al. 2021). With the presence of type III PKS genes in 70% of the known species (91 out of 131 planctomycetal genomes), these genes are found in a majority of the phylum, but are not conserved in all species.

**Fig. 1:**
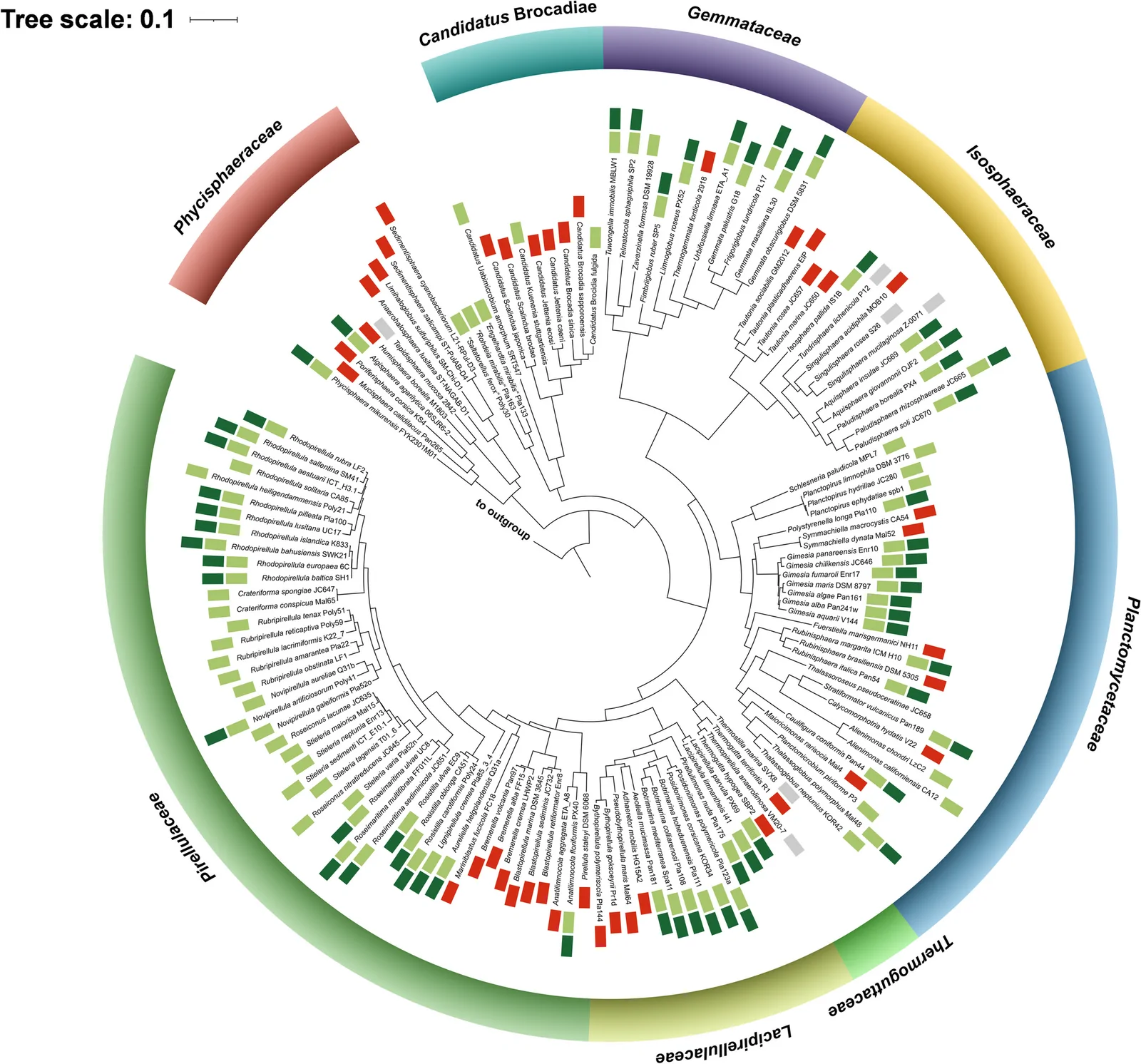
16S rRNA gene phylogenetic tree**.** A maximum likelihood phylogenetic tree was calculated based on the 16S rRNA gene sequences of the type strains of the currently described species within the phylum *Planctomycetota*. Sequences of three members of the PVC superphylum were used as outgroup (*Opitutus terrae*, NCBI acc. no. AJ229235; *Kiritimatiella glycovorans*, acc. no. NR_146840; and *Lentisphaera araneosa*, acc. no. NR_027571). The scale bar indicates the number of substitutions per nucleotide position. The presence of at least one type III polyketide synthase-encoding gene in the genome of a strain is indicated by a light green box next to the species name, whereas the absence of such genes is indicated by a red box. Gray boxes indicate species for which the genome has not been sequenced yet. A dark green box indicates the organization of the type III PKS gene in a three-gene operon with a methyltransferase and an oxidoreductase gene

As visualized in the phylogenetic tree, the presence or absence of type III PKS genes cannot be clearly attributed to the individual bacterial families (Fig. 1). However, their relative abundance is lower in the classes *Phycisphaerae* and *Candidatus* Brocadiae. This finding aligns with the observation that members of these classes have smaller genomes and harbor lower numbers of BGCs than members of the class *Planctomycetia* (Kallscheuer and Jogler 2021).

The encoded putative type III PKS proteins cover a size range from 274 aa (*Botrimarina mediterranea*, locus tag Spa11_43050) to 441 aa (*Rubripirellula amarantea*, locus tag Pla22_34060) and have an average size of 371 aa. All candidate sequences are either annotated as alpha-pyrone synthesis polyketide synthase, type III polyketide synthase, or chalcone and stilbene synthase domain-containing proteins. The compounds synthesized by these proteins are unknown. All planctomycetal type III PKSs harbor the *N*-terminal and *C*-terminal domains found in plant chalcone and stilbene synthases (Pfam domains PF00195 and PF02797).

**Fig. 2:**
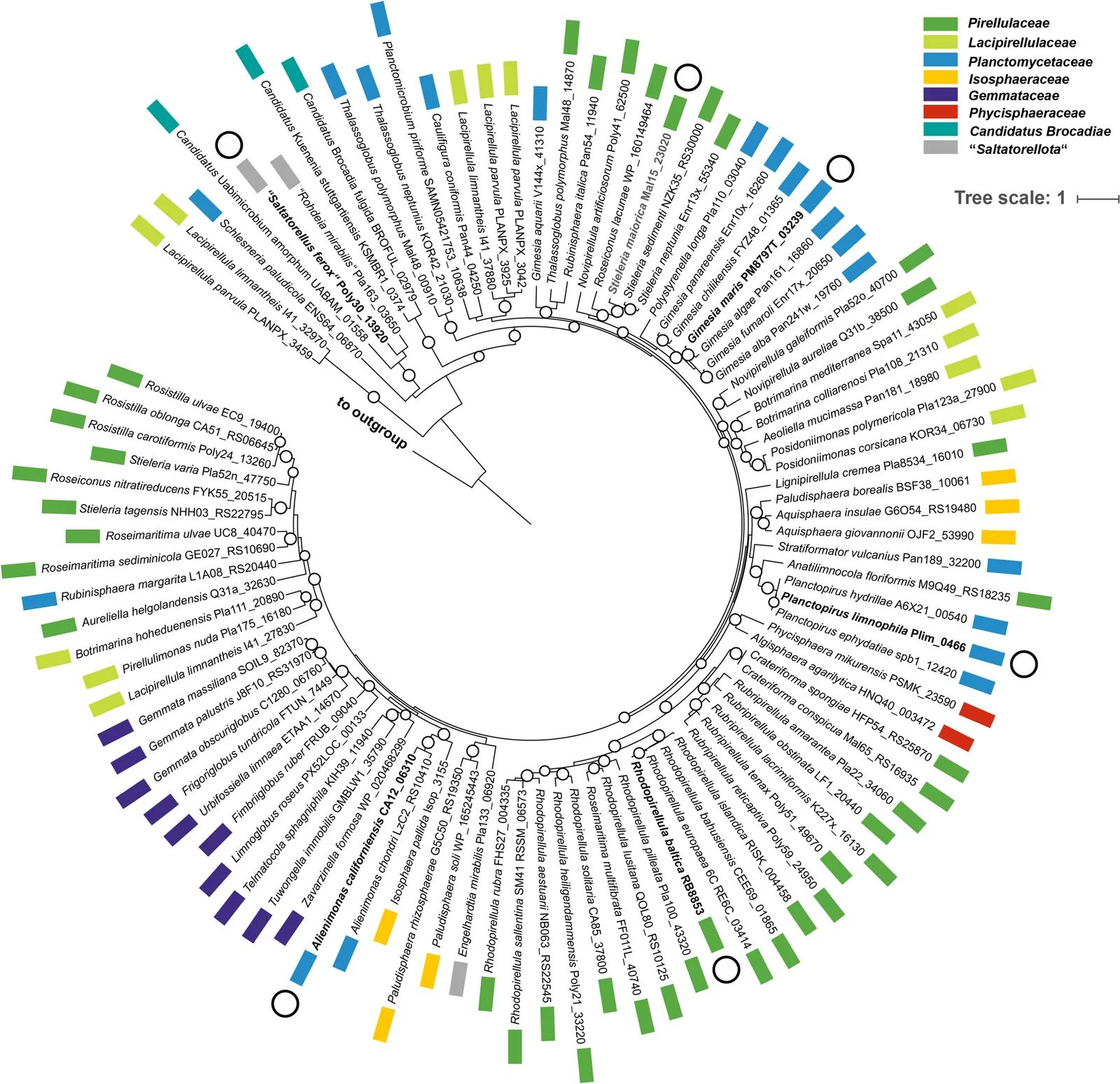
Phylogenetic tree based on type III polyketide synthase protein sequences. The maximum likelihood phylogenetic tree was calculated based on the protein sequences of all putative type III PKSs in members of the phylum *Planctomycetota*. The sequences of citrate synthase of *C. glutamicum* (Uniprot-ID P42457) and FtsZ of *E. coli* K12 (Uniprot-ID P0A9A6) were used as outgroup. Bootstrap values obtained after 1000 re-samplings were calculated, and values of > 90% are indicated by open circles on the branches. The circle size reflects the bootstrap values between 90% (smaller circles) and 100% (larger circles). Colored boxes next to the species name indicate members belonging to the same family or class. The larger open circles next to the species names indicate that the protein was selected for a heterologous expression of the respective gene in *C. glutamicum*

Of the analyzed set of 96 sequences, two did not cluster with all other sequences. These belong to the type strains of the two species that turned out to have three type III PKS genes (*L. parvula* PX69^T^ and *L. limnantheis* I41^T^) (Fig. 2, next to the outgroup). In a clustering analysis based on all-against-all BLAST + similarities, these two sequences did not cluster with any characterized type III PKS of bacterial, fungal, or plant origin (data not shown).

To gain more insights into the potential compound class produced by planctomycetal type III PKSs, we also analyzed the organization of neighboring genes encoded immediately up- and downstream. In 60% of the cases (58 of the 96 analyzed genes), the type III PKS gene turned out to be the last gene of a putative tricistronic operon, in which the PKS gene is preceded by a putative methyltransferase- and FAD-dependent oxidoreductase gene (Fig. 1, dark green boxes).

### Genetic organization of type III PKS genes and selection of candidates for heterologous expression experiments

Together with the phylogenetic distance of the donor strains, we considered the information on the genetic organization during selection of genes for the planned heterologous expression in *C. glutamicum*. Six putative type III PKSs from species belonging to different families were selected for a heterologous expression of the respective genes codon-optimized for *C. glutamicum*. The donor planctomycetes included the limnic model strain *Planctopirus limnophila* DSM 3776^ T^, the marine model strain *Rhodopirellula baltica* SH1^T^, the first isolated planctomycete strain *Gimesia maris* DSM 8797^ T^, and strains isolated within the recent years, *Stieleria maiorica* Mal15^T^, *Alienimonas californiensis* CA12^T^, and “*Saltatorellus ferox*” Poly30^T^ (a more distantly related strain probably belonging to a separate phylum). *S. maiorica* was included because of its genetical accessibility.

**Fig. 3:**
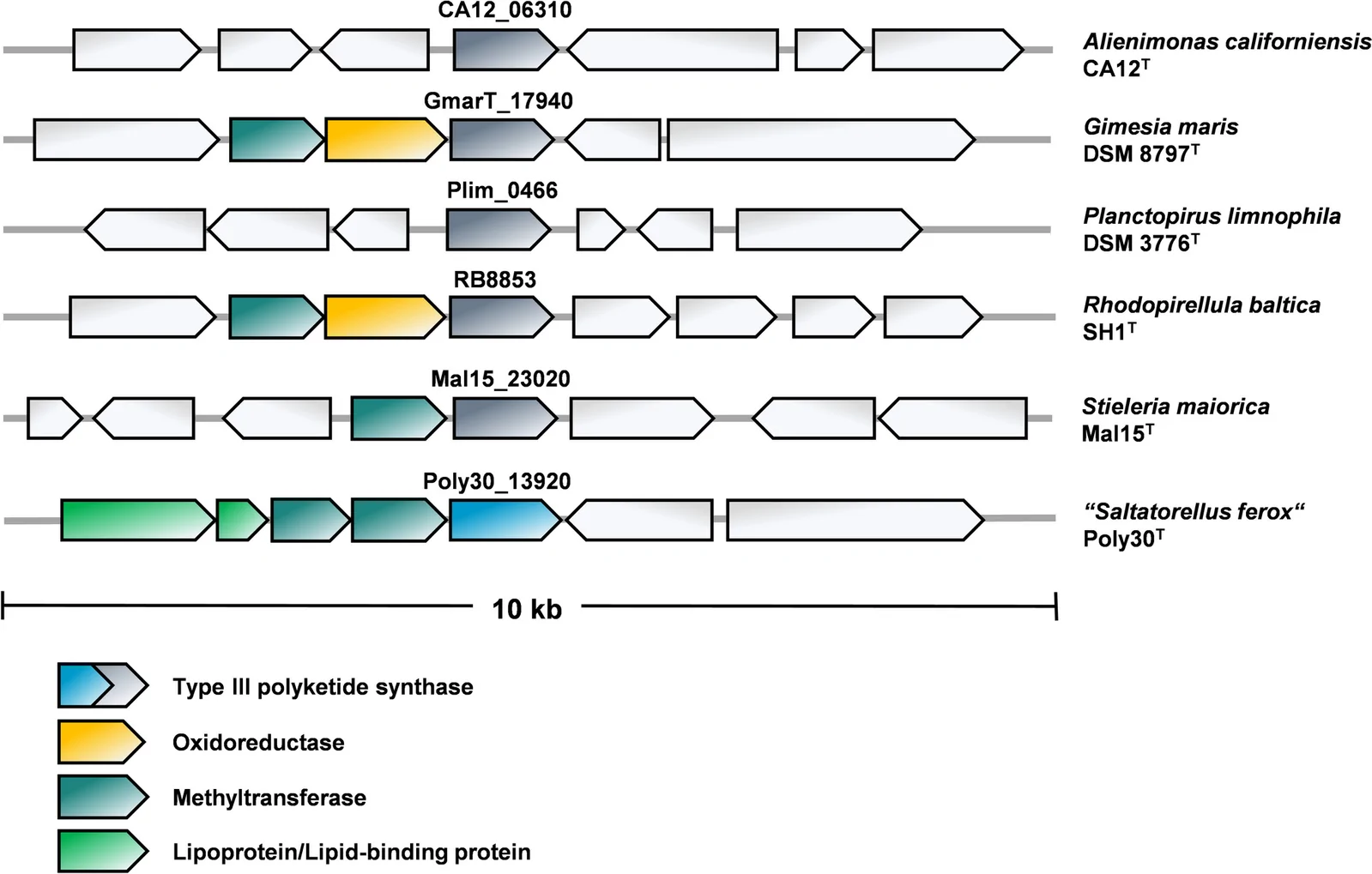
Genetic organization of the type III PKS genes selected for heterologous expression experiments in *C. glutamicum*. The type III PKS-encoding gene and the adjacent genes in six selected planctomycetal strains are depicted. Same colors indicate the same putative gene function: methyltransferase (turquoise), oxidoreductase (orange), type III polyketide synthase (grey-blue and blue, see text for details), putative lipoproteins (green). For the other genes (grey), the annotation is not specified here

An analysis of the genomic context showed that *G. maris* and *R. baltica* harbor the three gene operons (methyltransferase – oxidoreductase – type III PKS), whereas *S. maiorica* harbors a two-gene operon lacking the putative oxidoreductase gene (Fig. 3). In “*S. ferox*,” a putative pentacistronic operon was found. In this organism, the type III PKS gene is preceded by two putative methyltransferase-encoding genes that are in turn preceded by genes encoding a putative lipoprotein and lipid/isoprenoid-binding protein. The genes from *A. californiensis* and *P. limnophila* are probably not part of an operon, and neither methyltransferase nor oxidoreductase genes are encoded in direct proximity (Fig. 3).

Heterologous expression of planctomycetal genes in *C. glutamicum.*

The strain *C. glutamicum* M-CoA was used as a host for heterologous expression experiments (Milke et al. 2019, 2020). This strain was extensively engineered towards aromatic compound (in particular polyphenol) biosynthesis by the implementation of several genomic modifications (Milke et al. 2020), including the abolishment of all known catabolic pathways for aromatic compounds naturally present in the wild-type strain (Kallscheuer and Marienhagen 2018; Kallscheuer et al. 2016). In addition, the central metabolism of the strain was engineered to redirect the carbon flux towards malonyl-CoA, the rate-limiting metabolite for engineered biosynthesis of polyketide-derived phenolic compounds (Lim et al. 2011; Milke et al. 2019). The strain turned out to be suitable for the biosynthesis of polyketide-derived compounds from plants and fungi (Kallscheuer et al. 2019a, 2017). It also harbors the T7 RNA polymerase-encoding gene under the control of an IPTG-inducible promoter that allows the inducible expression of genes placed under the control of the strong T7 promoter (Kortmann et al. 2015). Genes coding for the six selected type III PKSs from planctomycetes (Fig. 3) were purchased as codon-optimized synthetic DNA fragments and cloned into the plasmid pMKEx2 allowing an expression under control of the strong T7 promoter.

**Fig. 4:**
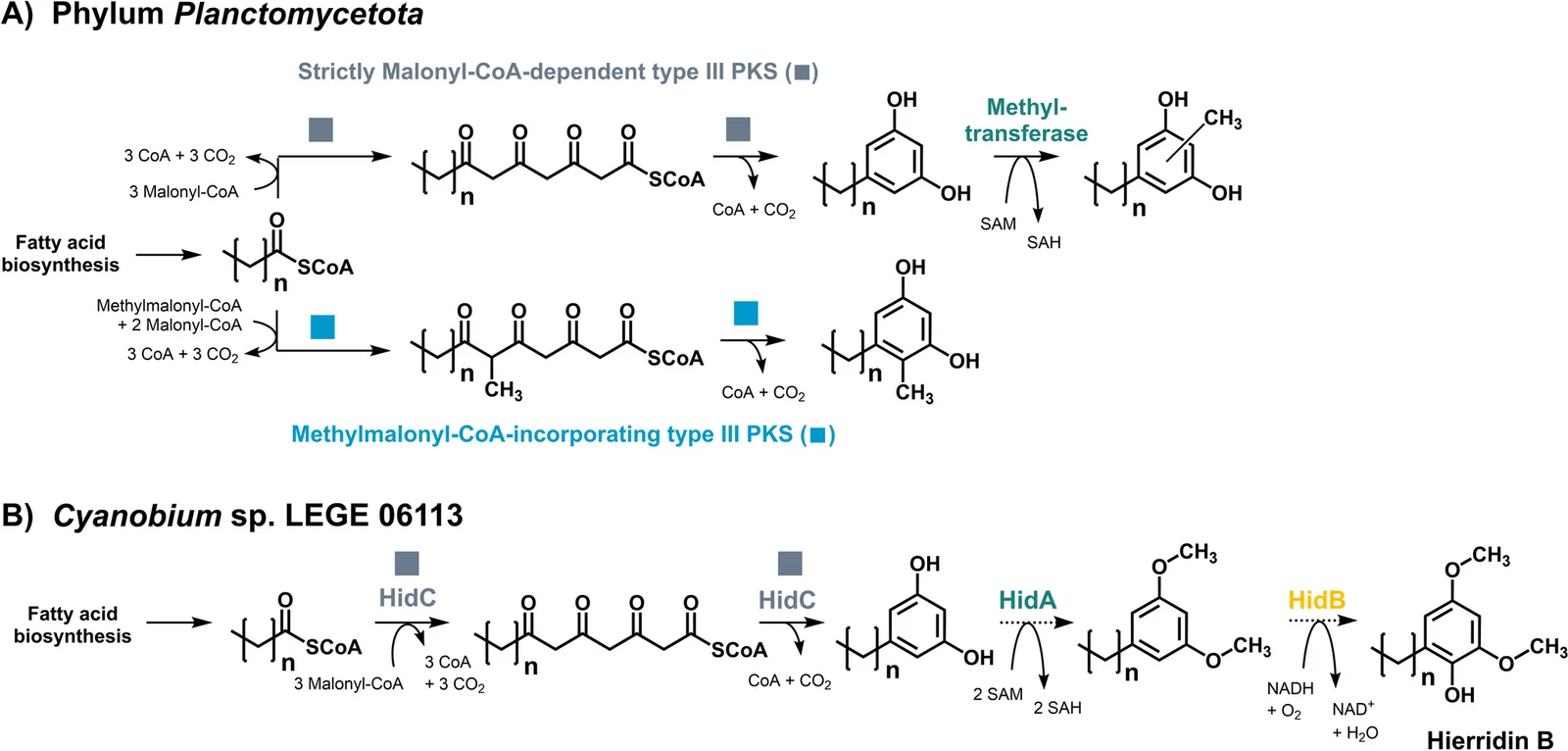
Postulated alkylresorcinol pathway in members of the phylum *Planctomycetota* and Hierridin biosynthesis pathway in *Cyanobium* sp. LEGE 06113. The reaction steps leading to alkylresorcinols including relevant co-substrates and products are depicted for the postulated alkylresorcinol pathway in members of the phylum *Planctomycetota*. The Hierridin biosynthetic pathway identified in *Cyanobium* sp. LEGE 06113 is shown for comparison. *n* = 14 or 16 for the planctomycetal pathway and *n* = 14 for the picocyanobacterial pathway. SAM, *S*-adenosyl methionine; SAH, *S*-adenosyl homocysteine

The constructed *C. glutamicum* strains, each expressing a single type III PKS gene, were cultivated in defined CGXII medium with 4% (w/v) glucose as the sole carbon and energy source under inducing conditions. An uninduced plasmid-free strain and a strain harboring the empty vectors (pMKEx2 and pEKEx3) were used as negative controls (Fig. S1A, B). All seven strains reached the same final OD_600_ of 42–45 after 72 h. The cultures were then harvested, and extracts were subjected to an untargeted GC-TOF–MS analysis. No products were detected for the episomal expression of the codon-optimized genes coding for the enzymes from *P. limnophila* (t3pks_*PlCg*_) and *A. californiensis* (t3pks_*AcCg*_) (that both do not harbor the three gene operons or variations thereof) (Fig. S1C, E). However, for the strains with enzymes from *G. maris* (t3pks_*GmCg*_), *R. baltica* (t3pks_*RbCg*_), and *S. maiorica* (t3pks_*SmCg*_), additional peaks not present in the empty vector control were detected (Fig. S1D, F, H). A comparison of the mass spectra against the NIST database and the in-house database JuPoD identified the compounds as alkylresorcinols (Table 3). Their identity was confirmed by virtual derivatization and electron impact (EI^+^) fragmentation of the predicted structure (Figs. S3-S8). Alkylresorcinols are derivatives of 1,3-dihydroxybenzene (resorcinol) that harbor an additional alkyl chain at C5 of the aromatic ring (in *meta* position to both hydroxy groups) (Fig. 4). This ring substitution pattern resembles compounds produced by other type III polyketide synthases, e.g., stilbene synthases (Parage et al. 2012).

**Table 3:** Characteristics of the alkylresorcinols detected using GC-TOF–MS analysis and information on the required precursors. *yes*, compound identified (EI^+^ spectrum and in silico fragmentation, see text for details); *n.d*., not detected

	**5-Pentadecyl-resorcinol**	**5-Heptadecyl-resorcinol**	**5-Hepta-decenyl-resorcinol**	**5-Penta-decyl-methyl-resorcinol**	**5-Hepta-decyl-methyl-resorcinol**	**5-Hepta-decenyl-methyl-resorcinol**
**Compound characteristics**						
Sum formula	C_21_H_36_O_2_	C_23_H_40_O_2_	C_23_H_38_O_2_	C_22_H_38_O_2_	C_24_H_42_O_2_	C_24_H_40_O_2_
Exact mass (g/mol)	320.27	348.30	346.29	334.29	362.32	360.30
Fatty acid starter unit	Palmitic acid (16:0)	Stearic acid (18:0)	Oleic acid (18:1)	Palmitic acid (16:0)	Stearic acid (18:0)	Oleic acid (18:1)
**Expressed gene(s)**						
t3pks_*AcCg*_	n.d.	n.d.	n.d.	n.d.	n.d.	n.d.
t3pks_*GmCg*_	yes	yes	yes	n.d.	n.d.	n.d.
t3pks_*GmCg*_ / mtr_*GmCg*_ + fdo_*GmCg*_	yes	yes	yes	yes	yes	yes
t3pks_*PlCg*_	n.d.	n.d.	n.d.	n.d.	n.d.	n.d.
t3pks_*RbCg*_	n.d.	yes	n.d.	n.d.	n.d.	n.d.
t3pks_*SmCg*_	yes	yes	n.d.	n.d.	n.d.	n.d.
t3pks_*SfCg*_	yes	yes	yes	yes	yes	yes
t3pks_Sf_ / mtr1_*SfCg*_ + mtr2_*SfCg*_	yes	yes	yes	yes	yes	yes

Based on the C2-C7 aldol condensation mechanism performed by stilbene synthases (Bhan et al. 2015), a putative biosynthesis pathway for the identified pentadecyl- and heptadecylresorcinols was outlined. In this pathway, the three alkylresorcinol-producing enzymes from *G. maris*, *R. baltica*, and *S. maiorica* use CoA-activated long-chain fatty acids as starter units and catalyze an iterative and decarboxylating condensation of three molecules of malonyl-CoA for production of a tetraketide intermediate that is subsequently cyclized and aromatized to a resorcinol residue (Fig. 4A, upper branch). CoA-activated stearic acid (18:0) and oleic acid (18:1*cis*-9) are the required starter units to produce 5-heptadecylresorcinol and 5-heptadecenylresorcinol, respectively (Table 3). The enzymes from *G. maris* and *S. maiorica* probably also accepted palmitoyl-CoA (16:0) as substrate to produce 5-pentadecylresorcinol. The three free fatty acids (16:0, 18:0, and 18:1*cis*-9) were also identified in the GC-TOF–MS analysis of the constructed *C. glutamicum* strains (Fig. S1). Hence, the suspected biosynthetic route appears plausible.

The enzyme from “*S. ferox*” also produced the three above-mentioned compounds (Fig. S1G); however, peaks with a similar fragmentation pattern in the EI^+^ spectrum but a mass increase of 14 Da were detectable. Moreover, in silico-fragmentation suggests an additional methyl group attached to the aromatic ring (Figs. S6-8). The most likely explanation for this observation is that the enzyme from “*S. ferox*” can incorporate the alternative extender unit methylmalonyl-CoA in one of the three chain elongation steps (Fig. 4A, lower branch).

The obtained results suggest that planctomycetal type III PKSs organized in the three gene operons (or variations thereof) act as alkylresorcinol synthases. To investigate the role of the other, in the natural host co-occurring genes, we constructed plasmids based on the vector pEKEx3 that is compatible with pMKEx2 in *C. glutamicum*. Codon-optimized genes encoding the putative FAD-dependent oxidoreductase (fdo) and methyltransferase (mtr) from *G. maris* were inserted as bicistronic operon into the multiple cloning site of pEKEx3 to yield pEKEx3_mtr_*GmCg*_-fdo_*GmCg*_ and the two putative methyltransferase genes from “*S. ferox*” were used for the construction of pEKEx3_mtr1_*SfCg*_-mtr2_*SfCg*_. Cultures of the strains *C. glutamicum* M-CoA pMKEx2_t3pks_*GmCg*_ pEKEx3_mtr_*GmCg*_-fdo_*GmCg*_ and *C. glutamicum* M-CoA pMKEx2_t3pks_*SfCg*_ pEKEx3_mtr1_*SfCg*_-mtr2_*SfCg*_ were prepared and processed as described for the previous experiment. The co-expression of the two additional genes derived from *G. maris* led to a single methylation in the alkylresorcinols (Fig. S2). The two hydroxy groups of the alkylresorcinols are shielded with trimethylsilyl (TMS) residues after compound derivatization prior to GC-TOF–MS analysis. Since only alkylresorcinol compounds with two TMS residues were identified, the additional methyl group is probably immediately connected to a carbon atom and not to an oxygen atom. However, the exact position of the *C*-methylation on the aromatic ring could not be determined from the fragmentation pattern. Taken together, the role of the putative FAD-dependent oxidoreductase from *G. maris* and the two methyltransferases from “*S. ferox*” Poly30^T^ remain to be elucidated. The identified alkylresorcinols produced by the planctomycetal type III PKSs are likely the first intermediate of an at least three-step pathway.

### Comparative analysis with bacterial and plant type III PKSs participating in explored biosynthesis pathways

**Fig. 5:**
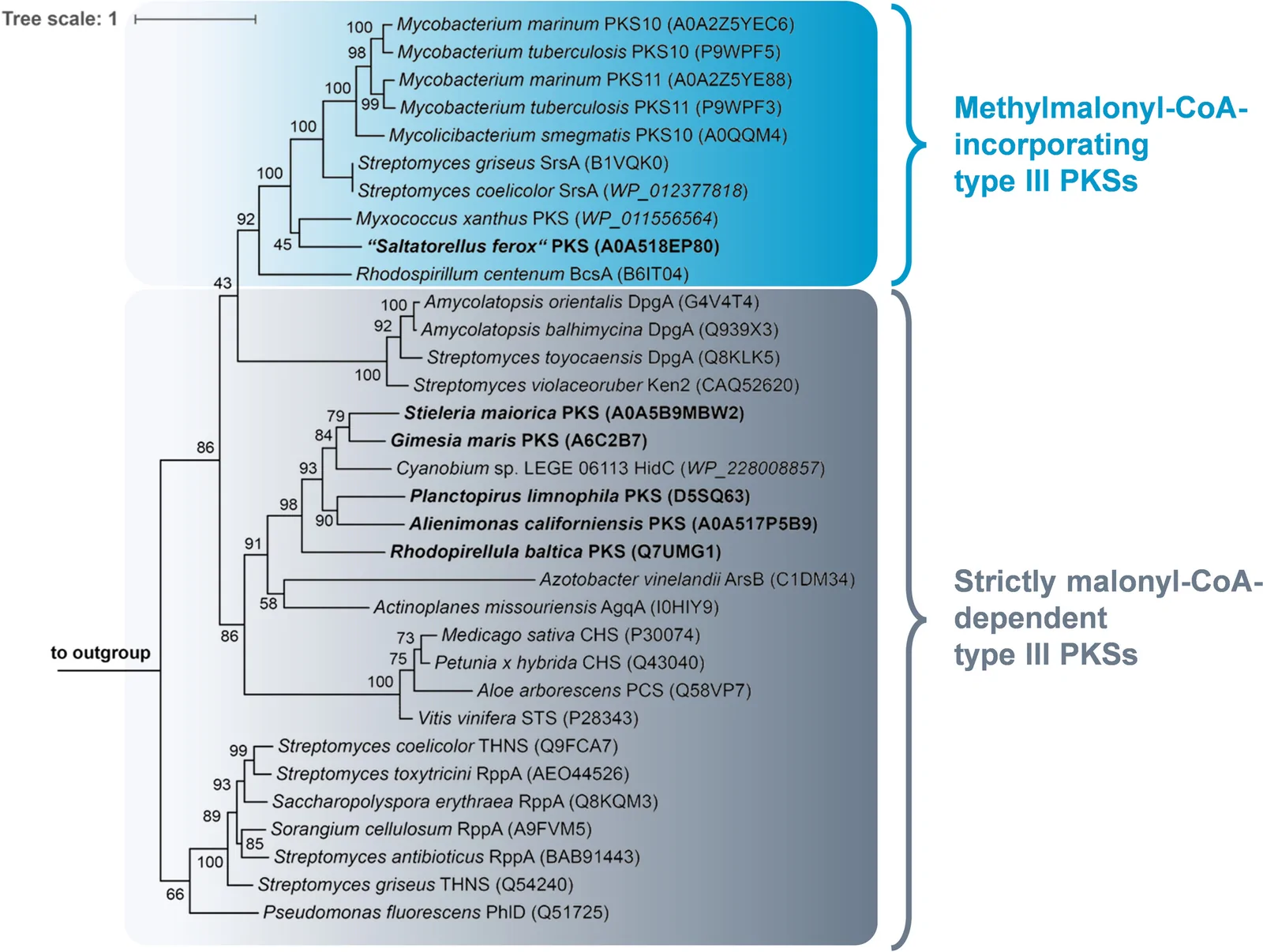
Phylogenetic tree of the investigated planctomycetal type III PKS and enzymes of this class with an identified reaction product from other organisms. The tree was constructed based on an alignment of type III PKS protein sequences. The sequences of citrate synthase of *C. glutamicum* (Uniprot-ID P42457) and FtsZ of *E. coli* K12 (Uniprot-ID P0A9A6) were used as outgroup. Bootstrap values obtained after 1000 re-sampling are shown on the nodes (in %). The Uniprot-ID of the used proteins are provided in brackets. In case that a protein was not listed in the Uniprot database, the NCBI accession numbers is presented alternatively (in italics)

To check for a potential protein sequence-function relationship, a maximum likelihood phylogenetic tree was constructed based on the six investigated planctomycetal enzymes and other plant or bacterial type III PKSs participating in already explored biosynthesis pathways. The type III PKSs used for comparison included model stilbene synthases (STS), chalcone synthases (CHS) and pentaketide chromone synthases (PCS) from plants, and synthases for 1,3,6,8-tetrahydroxynaphthalene (TNHS, RppA), phloroglucinol (PhlD), 3,5-dihydroxyphenylacetyl-CoA (DpgA), resorcinols (AgqA, SrsA), and α-pyrones (PKS11) from bacteria (Liu and Begley 2020). The clustering of type III PKSs (Fig. 5) reflects a possible sequence-function relationship as distinct clades were obtained for synthases that form the same compound classes. Similarly, methylmalonyl-CoA-incorporating PKSs did not cluster with their malonyl-CoA-specific counterparts. Five of the planctomycetal enzymes clustered within the clade of malonyl-CoA-specific enzymes, whereas the enzyme from “*S. ferox*” clustered within the clade of methylmalonyl-CoA-incorporating PKSs (Fig. 5). This clustering pattern is in line with the finding that (from the tested enzymes) only the type III PKS from “*S. ferox*” formed alkylresorcinols bearing an additional methyl group that potentially results from the incorporation of methylmalonyl-CoA in the growing polyketide chain. The other five tested planctomycetal synthases cluster on a separate branch with the type III PKS HidC from the picocyanobacterium *Cyanobium* sp. LEGE 06113 (Costa et al. 2019). The position of HidC in the phylogenetic tree was confirmed by the calculation of a protein sequence similarity matrix based on the alignment that was also used for tree construction (Table S2). Indeed, HidC showed higher protein sequence identity values to the enzymes from *G. maris* (54.8%) and *S. maiorica* (50.6%) than these two to the other tested planctomycetal type III PKSs (< 48%). The type III PKS HidC is not only an alkylresorcinol synthase that formed the same compounds as the closely related proteins tested from planctomycetes, but also the last gene in a three-gene operon preceded by a methyltransferase (HidA) and an oxidoreductase (HidB) (Costa et al. 2019).

### Analysis of potential functions of bacterial alkylresorcinols

Alkylresorcinols and decorated phenolic lipids derived thereof are amphiphilic compounds that have been identified across the bacterial kingdom (Zabolotneva et al. 2022). However, their natural functions are only occasionally understood. Cyanobacteria produce several alkylresorcinols with antibiotic activities (Martins et al. 2019). The actinomycete *Actinoplanes missouriensis* uses a type III PKS (AgqA) to produce alkylresorcinols, which are subsequently prenylated and hydroxylated (Awakawa et al. 2011); however, their function remains enigmatic. *Streptomyces griseus*, a member of the same phylum, and *Myxococcus xanthus* (class *Deltaproteobacteria*) further oxidize alkylresorcinols produced by type III PKSs (SrsA and FtpA, respectively) to alkylquinones (Funabashi et al. 2008; Hayashi et al. 2011). The inactivation of *srsA* in *S. griseus* led to an increased sensitivity to β-lactam antibiotics indicating that alkylquinones confer an increased resistance to this class of antibiotics (Funabashi et al. 2008). In *Mycobacterium tuberculosis*, alkylquinones (polyketide quinones) were shown to act as intermediate electron carriers during respiration in oxygen-deficient niches (Anand et al. 2015).

To deduce potential functions of alkylresorcinols in planctomycetes, the genetically accessible *S. maiorica* Mal15^T^ and the constructed *C. glutamicum* strains were subjected to further studies. An inactivation of the sole type III PKS-encoding gene in *S. maiorica* (locus tag Mal15_23020) was achieved by enforcing a simultaneous homologous recombination event up- and downstream of the open reading frame of Mal15_23020. The gene was exchanged for a heterologous DNA fragment that encodes a chloramphenicol acetyltransferase (*cat*) gene including its promoter. As a control, we also inactivated the gene Mal15_51000 that encodes a non-essential protein for which a deletion did not lead to a detectable phenotype under standard cultivation conditions. The recombinant strains *S. maiorica* ∆Mal15_23020::*cat* and *S. maiorica* ∆Mal15_51000::*cat* were cultivated in M3H NAG ASW medium with chloramphenicol; however, no differences in growth speed or final biomass yield were observed. Hence, alkylresorcinol biosynthesis is not essential in *S. maiorica* under the tested growth conditions.

To test for an influence of alkylresorcinol biosynthesis on the natural resistance of *S. maiorica* to β-lactam antibiotics, both strains were streaked on M3H NAG ASW agar with chloramphenicol and minimal inhibitory activity (MIC) test stripes with ampicillin (0.016–256 μg/mL) were placed on the plates. Both strains grew equally well up to the highest tested ampicillin concentration (Fig. S9). Hence, alkylresorcinols do not play a role as a resistance mechanism to β-lactam antibiotics in planctomycetes. This observation is in line with the presence of β-lactamases as resistance-conferring factors in planctomycetes and is additionally substantiated by the fact that also planctomycetal strains that lack type III PKS-encoding genes are resistant to β-lactam antibiotics. Similarly, cell material from the main cultures of the *C. glutamicum* strains producing planctomycetal alkylresorcinols was streaked on CGXII agar with 4% glucose (w/v), 1 mM IPTG and the respective antibiotics for plasmid maintenance (kanamycin and/or spectinomycin). Ampicillin MIC test stripes yielded similar MICs between 1.5 and 2 µg/mL for all tested strains and provided no indications for an increased resistance (Fig. S10). Since the heterologous planctomycetal enzymes did not oxidize the produced compounds to alkylquinones in *C. glutamicum*, a similar effect as observed for alkylquinones in *S. griseus* cannot be excluded at this stage. It should also be noted that the alkylresorcinols found in the constructed *C. glutamicum* strains are not necessarily the identical (final) compounds produced in the planctomycetes.

Culture extracts of the constructed *C. glutamicum* strains were used for agar disc diffusion assays to test for antimicrobial activities. Unexpectedly, zones of inhibition were obtained for all cultures including the empty vector control (Fig. S11). However, these resulted from the toxicity of the inducer IPTG in the concentrated extract. An additional antimicrobial effect of the alkylresorcinol core structures formed in *C. glutamicum* was not observed. The exact function of the compounds remains elusive.

## Discussion

Bioinformatic genome mining of the current open collection of 131 planctomycetal species with a sequenced genome yielded 96 putative type III PKS genes. The phylogenetic tree based on the encoded protein sequences did not strictly reflect the phylogenetic relationship of the strains to which they belong (Fig. 2). However, proteins from species belonging to the same genus clustered in most cases. It has been reported that the phylogeny of biosynthetic enzymes shows a cluster pattern according to their catalytic function and rapidly deviates from the phylogeny of their host strains (Medema et al. 2014), explaining the observed low resolution of the type III PKS protein sequences when used as phylogenetic marker.

Fifty-eight of the analyzed strains harbor a conserved three-gene operon consisting of a putative methyltransferase-, an oxidoreductase-, and a type III PKS-encoding gene. The cluster is spread across different families within the phylum and strains from various habitats (freshwater, seawater, and soil). An exemplary empirical analysis of four of these clusters (two of them with variations of the three gene operons) confirms their relevance for the biosynthesis of alkylresorcinols. The type III PKSs of all tested strains except “*S. ferox*” clustered with HidC of *Cyanobium* sp. LEGE 06113. The gene *hidC* is part of the more recently identified three-gene operon *hidABC* that is responsible for the production of hierridin B, a 5-pentadecylresorcinol derivative that is additionally hydroxylated at position 4 and *O*-methylated at positions 1 and 3 of the phenolic residue (Costa et al. 2019) (Fig. 4B). Hierridins were already identified in the early 1990s in the lichen *Ramalina farinacea* and in the cyanobacterium *Phormidium ectocarpi* (Gonzalez et al. 1992; Papendorf et al. 1998). Ten years ago, additional hierridins were discovered in filamentous and unicellular marine cyanobacteria and anti-tumor activities were reported (Leão et al. 2013). Hierridin B was recently used as a basis to synthesize chemical analogues that turned out to have potent anti-proliferative activity (Brandão et al. 2020).

The *hidABC* cluster is found in many cyanobacterial genomes, and a close relationship to the here investigated cluster in members of the phylum *Planctomycetota* was already observed by the authors of the hierridin study. The order of the genes in the operon (methyltransferase–oxidoreductase–type III PKS) is conserved in nearly all analyzed planctomycetes in which the operon is present and identical to the cyanobacterial *hidABC* operon. On protein level, all three encoded proteins show a similar degree of sequence identity between 48 and 55% to the cyanobacterial counterparts. While the planctomycetal type III polyketide synthases synthesized the same major product as HidC, the specificity of the methyltransferases differs. The enzyme of *G. maris* is probably a *C*-methyltransferase, whereas HidA methylates the two phenolic hydroxy groups of the alkylresorcinol (Costa et al. 2019). The high degree of relationship in terms of genetic organization, enzymatic activity, and protein sequence identity raises the question if the cluster was transferred from one phylum to the other via horizontal gene transfer (Meslet-Cladière et al. 2013). The phyla *Cyanobacteriota* and *Planctomycetota* are only distantly related (Hug et al. 2016); hence, it appears rather unlikely that the cluster was already present in a common ancestor. The horizontal gene transfer hypothesis receives further support when considering that planctomycetes are often associated with aquatic phototrophs (Kallscheuer et al. 2021; Lage and Bondoso 2014). Genes up- and downstream of the planctomycetal operon are not conserved compared to *Cyanobium* sp. LEGE 06113. Adjacent genes are also not conserved among the analyzed planctomycetal genomes, but only in close relatives of a respective strain. The operon is widely distributed in cyanobacteria, and the analyzed *Cyanobium* sp. (or an ancestor) is hence not necessarily the donor/acceptor of the transfer event. The hypothesis further substantiates suspected planctomycete-cyanobacterial allelopathic interactions and thus deserves further attention.

Since the recombinant *C. glutamicum* strains only produced the alkylresorcinol core, but not the fully decorated compounds suspected in planctomycetes, an elucidation of the natural function is hampered by current limitations of the used heterologous expression approach. Still, the acquired knowledge on the chemical nature of the identified compounds allows for a targeted search in planctomycetal extracts in future studies. The results also indicate that alkylresorcinols are not the only compounds produced by planctomycetal type III PKS. Structurally more unrelated enzymes in strains harboring more than one type III PKS and genes not part of the characterized operon are probably only a small fraction of the yet for the most part untapped chemical repertoire of the phylum *Planctomycetota*. Bioprospection in the phylum will be guided by the continuous exploration of the phylogenetic diversity and the development of more sophisticated genetic tools.

## Supplementary Information

Below is the link to the electronic supplementary material.

Supplementary file1 (PDF 3144 KB)

## Data Availability

The datasets generated during and/or analyzed during the current study are available from the corresponding author on reasonable request.
