# Supplementary material for: A type III polyketide synthase cluster in the phylum Planctomycetota is involved in alkylresorcinol biosynthesis
Source: Appl Microbiol Biotechnol. 2024 Feb 26;108:239. doi: 10.1007/s00253-024-13065-x (PMC10896814; doi:10.1007/s00253-024-13065-x)
Supplement: Supplementary file 1 [file 253_2024_13065_MOESM1_ESM.pdf]

Supplementary Materials

A type III polyketide synthase cluster in the phylum *Planctomycetota* is involved in alkylresorcinol biosynthesis

Lars Milke[1], Moses Kabuu[2], Renè Zschoche[2], Jochem Gätgens[1], Karin Krumbach[1], Kim-Loreen Carlstedt[2], Carmen E. Wurzbacher[2], Sven Balluff[3], Christine Beemelmanns[3,4], Christian Jogler[2], Jan Marienhagen[1,5] and Nicolai Kallscheuer[2,#]

[1] Institute of Bio- and Geosciences, IBG-1: Biotechnology, Forschungszentrum Jülich, 52425 Jülich, Germany

[2] Department of Microbial Interactions, Institute for Microbiology, Friedrich Schiller University, 07743 Jena, Germany

[3] Helmholtz Institute for Pharmaceutical Research Saarland (HIPS), 66123 Saarbrücken, Germany

[4] Saarland University, Saarbrücken, Germany

[5] Institute of Biotechnology, RWTH Aachen University, Worringer Weg 3, 52074 Aachen, Germany

[#]Corresponding author:

Nicolai Kallscheuer, phone +49 3641 949337, e-mail nicolai.kallscheuer@uni-jena.de

Running Title: Akylresorcinol production by planctomycetal type III polyketide synthases

Keywords:
planctomycetes, *Corynebacterium glutamicum*, polyketide-derived compounds, hierridin, chemical mediators

Table S1. GenBank accession numbers of the analysed reference genomes

GenBank Acc. No.	Species name	Type strain
GCA_007741515.1	*Adhaeretor mobilis*	HG15A2
GCA_007748035.1	*Aeoliella mucimassa*	Pan181
GCA_014207595.1	*Algisphaera agarilytica*	DSM 103725
GCA_007743815.1	*Alienimonas californiensis*	CA12
GCA_013036045.1	*Alienimonas chondri*	LzC2
GCA_002007645.1	*Anaerohalosphaera lusitana*	ST-NAGAB-D1
GCA_007747655.1	*Anatilimnocola aggregata*	ETA_A8
GCA_024256385.1	*Anatilimnocola floriformis*	PX40
GCA_008087625.1	*Aquisphaera giovannonii*	OJF2
GCA_011064615.1	*Aquisphaera insulae*	JC669
GCA_007752135.1	*Aureliella helgolandensis*	Q31a
GCA_000153105.1	*Blastopirellula marina*	DSM 3645
GCA_007859755.1	*Blastopirellula retiformator*	Enr8
GCA_020966695.1	*Blastopirellula sediminis*	JC733
GCA_007859955.1	*Botrimarina colliarenosi*	Pla108
GCA_007859815.1	*Botrimarina hoheduenensis*	Pla111
GCA_007753265.1	*Botrimarina mediterranea*	Spa11
GCA_013618625.1	*Bremerella alba*	FF15
GCA_003335485.1	*Bremerella cremea*	HEX-1 MGV
GCA_007748115.1	*Bremerella volcania*	Pan97
GCA_008065115.1	*Bythopirellula goksoeyrii*	Pr1d
GCA_007860015.1	*Bythopirellula polymerisocia*	Pla144
GCA_007745435.1	*Calycomorphotria hydatis*	V22
GCA_001753675.2	*Candidatus* Brocadia sapporoensis	40
GCA_000949635.1	*Candidatus* Brocadia sinica	JPN1
GCA_000987375.1	*Candidatus* Brocadia fulgida	RU1
GCA_013360885.1	*Candidatus* Jettenia caeni	MAG_9
GCA_005524015.1	*Candidatus* Jettenia ecosi	J2
GCA_011066545.1	*Candidatus* Kuenenia stuttgartiensis	CSTR1
GCA_000786775.1	*Candidatus* Scalindua brodae	RU1
GCA_002443295.1	*Candidatus* Scalindua japonica	husup-a2
GCA_009002475.1	*Candidatus* Uabimicrobium amorphum	SRT547
GCA_007745175.1	*Caulifigura coniformis*	Pan44
GCA_007752935.1	*Crateriforma conspicua*	Mal65
GCA_012290005.1	*Crateriforma spongiae*	JC647
GCA_007748095.1	*Engelhardtia mirabilis*	Pla133
GCA_002197845.1	*Fimbriiglobus ruber*	SP5
GCA_013128195.2	*Frigoriglobus tundricola*	PL17
GCA_001983935.1	*Fuerstiella marisgermanici*	NH11
GCA_901538265.1	*Gemmata massiliana*	Soil9
GCA_901538385.1	*Gemmata obscuriglobus*	UQM 2246
GCA_017939745.1	*Gemmata palustris*	G18
GCA_007744675.1	*Gimesia alba*	Pan241w
GCA_007746795.1	*Gimesia algae*	Pan161
GCA_007748195.1	*Gimesia aquarii*	V144

GenBank Acc. No.	Species name	Type strain
GCA_008329715.1	*Gimesia chilikensis*	JC646
GCA_007754425.1	*Gimesia fumaroli*	Enr17
GCA_007747015.1	*Gimesia maris*	CA11
GCA_007748015.1	*Gimesia panareensis*	Pan110
GCA_015169395.1	*Humisphaera borealis*	M1803
GCA_000186345.1	*Isosphaera pallida*	ATCC 43644
GCA_007746075.1	*Lacipirellula limnantheis*	I41
GCA_009177095.1	*Lacipirellula parvula*	PX69
GCA_007751035.1	*Lignipirellula cremea*	Pla85_3_4
GCA_001999965.1	*Limihaloglobus sulfuriphilus*	SM-Chi-D1
GCA_008254045.1	*Limnoglobus roseus*	PX52
GCA_007747795.1	*Maioricimonas rarisocia*	Mal4
GCA_008087665.1	*Mariniblastus fucicola*	FC18
GCA_007748075.1	*Mucisphaera calidilacus*	Pan265
GCA_007860135.1	*Novipirellula artificiosorum*	Poly41
GCA_007860185.1	*Novipirellula aureliae*	Q31b
GCA_007860095.1	*Novipirellula galeiformis*	Pla52o
GCA_001956985.1	*Paludisphaera borealis*	PX4
GCA_011065895.1	*Paludisphaera rhizosphaereae*	JC665
GCA_011064595.1	*Paludisphaera soli*	JC670
GCA_000284115.1	*Phycisphaera mikurensis*	NBRC 102666
GCA_000025185.1	*Pirellula staleyi*	DSM 6068
GCA_007750855.1	*Pirellulimonas nuda*	Pla175
GCA_900113665.1	*Planctomicrobium piriforme*	DSM 26348
GCA_007752345.1	*Planctopirus ephydatiae*	spb1
GCA_001707835.1	*Planctopirus hydrillae*	JC280
GCA_000092105.1	*Planctopirus limnophila*	DSM 3776
GCA_007750395.1	*Polystyrenella longa*	Pla110
GCA_007747445.1	*Poriferisphaera corsica*	KS4
GCA_007859765.1	*Posidoniimonas corsicana*	KOR34
GCA_007859935.1	*Posidoniimonas polymericola*	Pla123a
GCA_007859945.1	*Pseudobythopirellula maris*	Mal64
GCA_023701485.1	*Rhodopirellula aestuarii*	ICT_H3.1
GCA_002727185.1	*Rhodopirellula bahusiensis*	SWK21
GCA_000196115.1	*Rhodopirellula baltica*	SH1
GCA_000346315.1	*Rhodopirellula europaea*	SH398
GCA_007860105.1	*Rhodopirellula heiligendammensis*	Poly21
GCA_001027925.1	*Rhodopirellula islandica*	K833
GCA_900182915.1	*Rhodopirellula lusitana*	DSM 25457
GCA_007859915.1	*Rhodopirellula pilleata*	Pla100
GCA_014192335.1	*Rhodopirellula rubra*	CECT 8075
GCA_000346505.1	*Rhodopirellula sallentina*	SM41
GCA_007859855.1	*Rhodopirellula solitaria*	CA85
GCA_007750655.1	*Rohdeia mirabilis*	Pla163
GCA_008312935.1	*Roseiconus lacunae*	JC635
GCA_008629675.1	*Roseiconus nitratireducens*	JC645
GCA_007741495.1	*Roseimaritima multifibrata*	FF011L
GCA_009618275.1	*Roseimaritima sediminicola*	JC651

GenBank Acc. No.	Species name	Type strain
GCA_008065135.1	*Roseimaritima ulvae*	UC8
GCA_007753095.1	*Rosistilla carotiformis*	Poly24
GCA_007751715.1	*Rosistilla oblonga*	CA51
GCA_007741475.1	*Rosistilla ulvae*	EC9
GCA_000165715.3	*Rubinisphaera brasiliensis*	DSM 5305
GCA_007859715.1	*Rubinisphaera italica*	Pan54
GCA_022267515.1	*Rubinisphaera margarita*	ICM_H10
GCA_007859865.1	*Rubripirellula amarantea*	Pla22
GCA_007741535.1	*Rubripirellula lacrimiformis*	K22.7
GCA_008374075.1	*Rubripirellula obstinata*	LF1
GCA_007860175.1	*Rubripirellula reticaptiva*	Poly59
GCA_007860125.1	*Rubripirellula tenax*	Poly51
GCA_007751475.1	*Saltatorellus ferox*	Poly30
GCA_000255655.1	*Schlesneria paludicola*	DSM 18645
GCA_001997385.1	*Sedimentisphaera cyanobacteriorum*	L21-RPul-D3
GCA_002117005.1	*Sedimentisphaera salicampi*	ST-PulAB-D4
GCA_000242455.3	*Singulisphaera acidiphila*	DSM 18658
GCA_008035925.1	*Stieleria maiorica*	Mal15
GCA_007754155.1	*Stieleria neptunia*	Enr13
GCA_025060895.1	*Stieleria sedimenti*	ICT_E10.1
GCA_024129865.1	*Stieleria tagensis*	TO1_6
GCA_007860045.1	*Stieleria varia*	Pla52n
GCA_007744515.1	*Stratiformator vulcanicus*	Pan189
GCA_007747995.1	*Symmachiella dynata*	Mal52
GCA_007860075.1	*Symmachiella macrocystis*	CA54
GCA_009177065.1	*Tautonia marina*	JC650
GCA_007752535.1	*Tautonia plasticadhaerens*	ElP
GCA_012958305.1	*Tautonia rosea*	JC657
GCA_003977685.1	*Tautonia sociabilis*	GM2012
GCA_018398935.1	*Telmatocola sphagniphila*	SP2
GCA_007859735.1	*Thalassoglobus neptunius*	KOR42
GCA_007744255.1	*Thalassoglobus polymorphus*	Mal48
GCA_011634775.1	*Thalassoroseus pseudoceratinae*	JC658
GCA_013694095.1	*Thermogemmata fonticola*	2918
GCA_002277955.1	*Thermogutta terrifontis*	R1
GCA_901538355.1	*Tuwongella immobilis*	MBLW1
GCA_007747215.1	*Urbifossiella limnaea*	ETA_A1
GCA_000255705.1	*Zavarzinella formosa*	DSM 19928

Table S2. Protein sequence similarity matrix of type III PKSs from planctomycetes and *Cyanobium* sp. LEGE 06113. The similarity matrix was calculated from the same alignment, which was also used for the construction of the phylogenetic tree presented in Figure 5. Identity values are given in %.

**Species (type III PKS gene locus tag)**	*Stieleria maiorica* (Mal15_23020)	*Rhodopirellula baltica* (RB8853)	*Gimesia maris* (PM8797T_03239)	*Alienimonas californiensis* (CA12_06310)	*Planctopirus limnophila* (Plim_0466)	"*Saltatorellus ferox*" (Poly30_13920)	*Cyanobium* sp. LEGE 06113 (IQ220_04565)
Stieleria maiorica (Mal15_23020)	100.0	42.7	57.7	46.7	45.2	33.0	50.6
Rhodopirellula baltica (RB8853)	42.7	100.0	43.5	40.0	40.3	28.2	43.8
Gimesia maris (PM8797T_03239)	57.7	43.5	100.0	47.9	46.5	30.3	54.8
Alienimonas californiensis (CA12_06310)	46.7	40.0	47.9	100.0	47.9	31.5	48.7
Planctopirus limnophila (Plim_0466)	45.2	40.3	46.5	47.9	100.0	27.9	47.4
"*Saltatorellus ferox*" (Poly30_13920)	33.0	28.2	30.3	31.5	27.9	100.0	33.2
Cyanobium sp. LEGE 06113 (IQ220_04565)	50.6	43.8	54.8	48.7	47.4	33.2	100.0

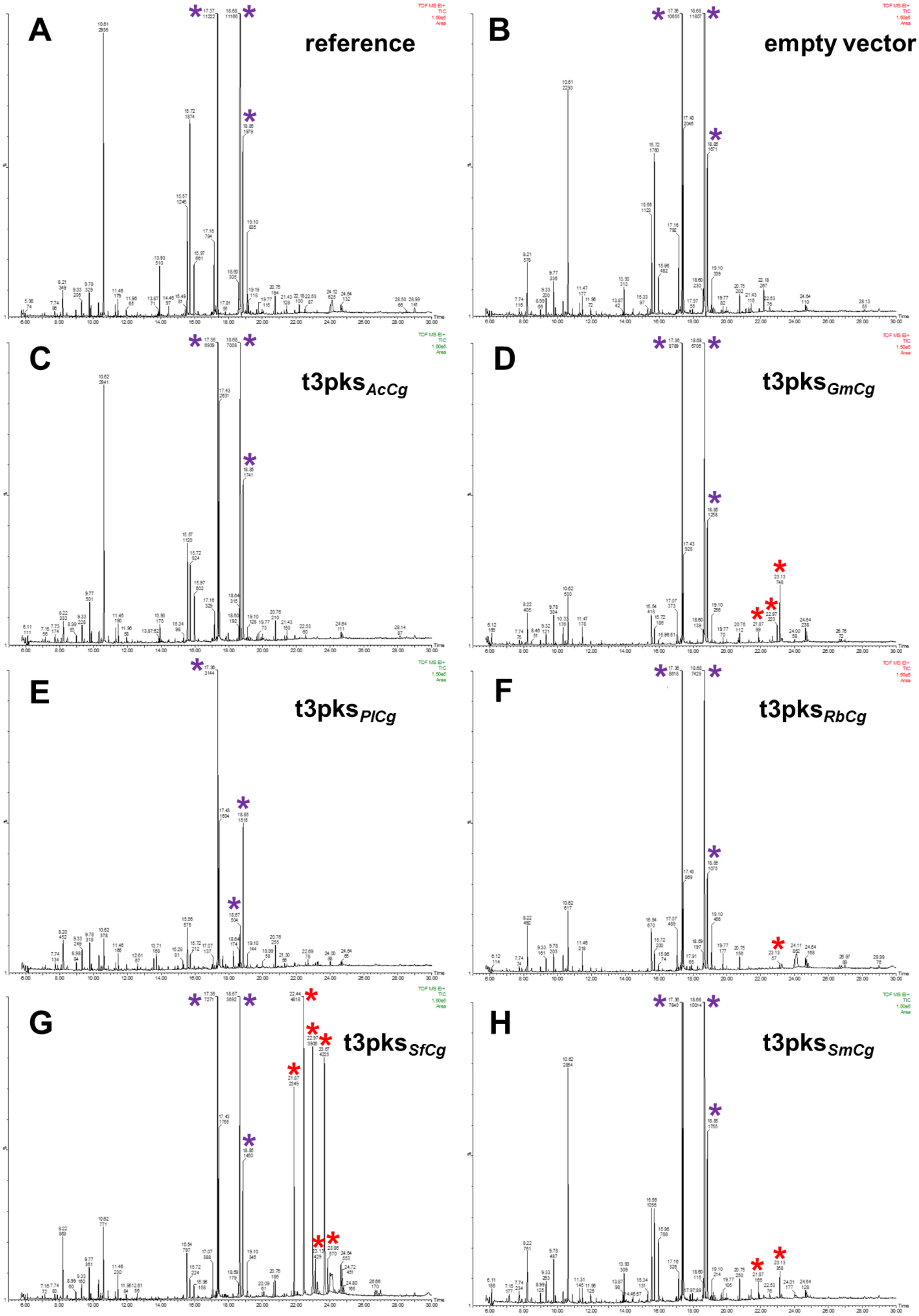
A
reference
B
empty vector
C
t3pks$_{AcCg}$
D
t3pks$_{GmCg}$
E
t3pks$_{PlCg}$
F
t3pks$_{RbCg}$
G
t3pks$_{SfCg}$
H
t3pks$_{SmCg}$

◁ **Figure S1. Total ion chromatograms of extracts from the *C. glutamicum* strains expressing genes encoding planctomycetal type III PKSs.** The used *C. glutamicum* strain without plasmid and without induction (reference) and the IPTG-induced strain carrying pMKEx2 (empty vector) served as negative controls. Peaks corresponding to fatty acids acting as starter units for the type III PKS-catalyzed reactions are highlighted with purple asterisks. Red asterisks denote peaks that correspond to the determined alkylresorcinols. Peak annotations: 17.37 min: palmitate [hexadecanoate] (1 TMS) RI 2044, 18.68 min: oleate [octadecenoate-9-*cis*] (1 TMS) RI 2215, 18.85 min stearate [octadecanoate] (1 TMS) RI 2239, 21.87 min: 5-pentadecyl-resorcinol (2 TMS) RI 2692, 22.44 min: 5-pentadecyl-methyl-resorcinol (2 TMS) RI 2784, 22.97 min: 5-heptadecenyl-resorcinol (2 TMS) RI 2858, 23.13 min: 5-heptadecyl-resorcinol (2 TMS) RI 2880, 23.67 min: 5-heptadecenyl-methyl-resorcinol (2 TMS) RI 2953, 23.86 min: 5-heptadecyl-methyl-resorcinol (2 TMS) RI 2979. Abbreviations: *Ac*, *Alienimonas californiensis*; *Cg*, *Corynebacterium glutamicum*, *Gm*, *Gimesia maris*, *Pl*: *Planctopirus limnophila*, *Rb*: *Rhodopirellula baltica*; *Sf*, *Saltatorellus ferox*; *Sm*, *Stieleria maiorica*; RI, retention index; TMS, trimethylsilyl resdiues.

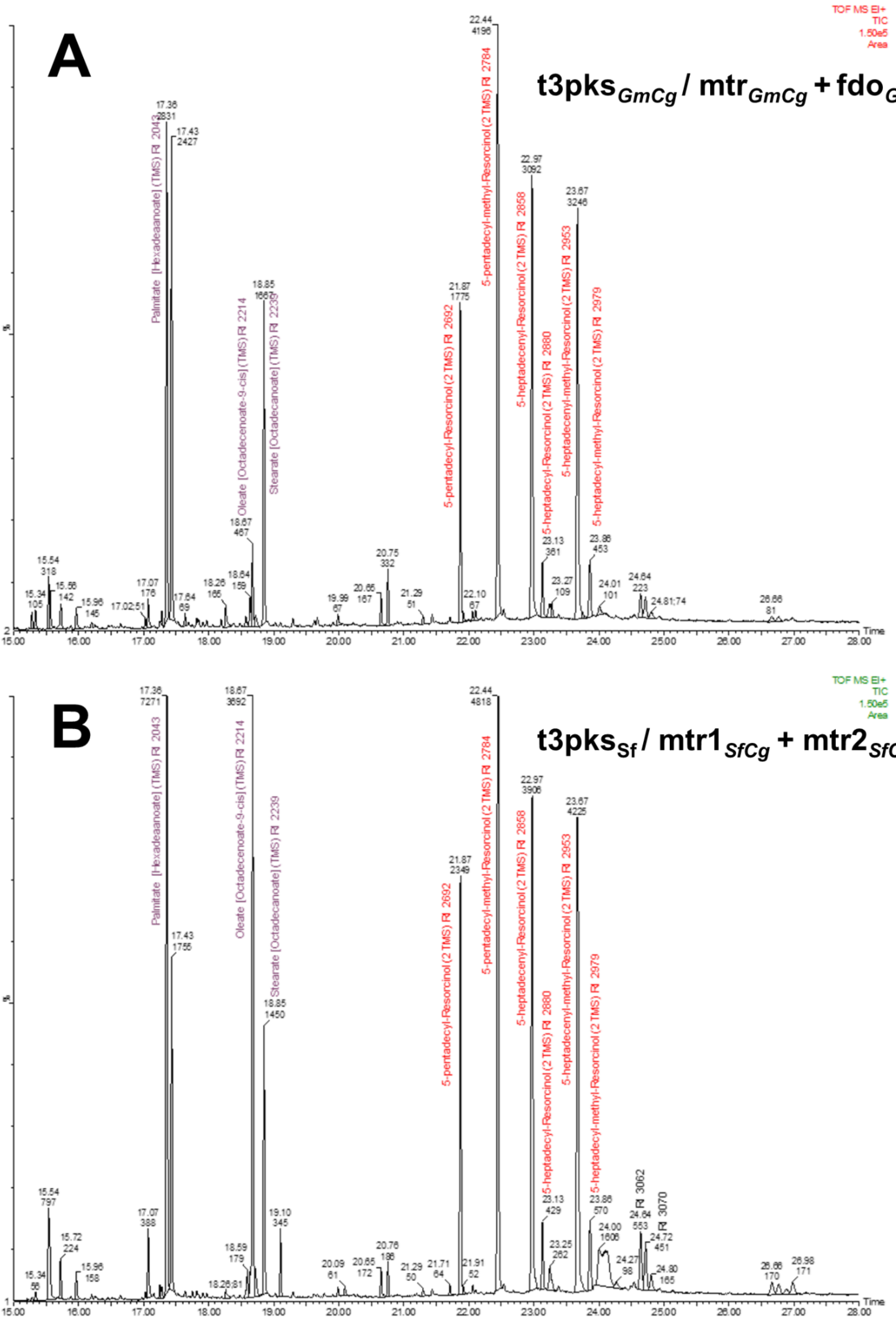
A
t3pks$_{GmCg}$ / mtr$_{GmCg}$ + fdo$_{GmCg}$
TOF MS EI+
TIC
1.50e5
Area
Palmitate [Hexadeaanoate] (TMS) RI 2043
Oleate [Octadecenoate-9-cis] (TMS) RI 2214
Stearate [Octadecanoate] (TMS) RI 2239
5-pentadecyl-Resorcinol (2 TMS) RI 2692
5-pentadecyl-methyl-Resorcinol (2 TMS) RI 2784
5-heptadecenyl-Resorcinol (2 TMS) RI 2858
5-heptadecyl-Resorcinol (2 TMS) RI 2880
5-heptadecenyl-methyl-Resorcinol (2 TMS) RI 2953
5-heptadecyl-methyl-Resorcinol (2 TMS) RI 2979
Time
B
t3pks$_{Sf}$ / mtr1$_{SfCg}$ + mtr2$_{SfCg}$
TOF MS EI+
TIC
1.50e5
Area
Palmitate [Hexadeaanoate] (TMS) RI 2043
Oleate [Octadecenoate-9-cis] (TMS) RI 2214
Stearate [Octadecanoate] (TMS) RI 2239
5-pentadecyl-Resorcinol (2 TMS) RI 2692
5-pentadecyl-methyl-Resorcinol (2 TMS) RI 2784
5-heptadecenyl-Resorcinol (2 TMS) RI 2858
5-heptadecyl-Resorcinol (2 TMS) RI 2880
5-heptadecenyl-methyl-Resorcinol (2 TMS) RI 2953
5-heptadecyl-methyl-Resorcinol (2 TMS) RI 2979
RI 3062
RI 3070
Time

◁ **Figure S2. Total ion chromatograms of extracts from the *C. glutamicum* strains co-expressing genes encoding type III PKSs and hydroxylases/methyltransferases.** Peaks corresponding to fatty acids acting as starter units for the type III PKS-catalyzed reactions are highlighted with purple asterisks. Red asterisks denote peaks that correspond to the determined alkylresorcinols. Peak annotations: 17.37 min: palmitate [hexadecanoate] (1 TMS) RI 2044, 18.68 min: oleate [octadecenoate-9-*cis*] (1 TMS) RI 2215, 18.85 min stearate [octadecanoate] (1 TMS) RI 2239, 21.87 min: 5-pentadecyl-resorcinol (2 TMS) RI 2692, 22.44 min: 5-pentadecyl-methyl-resorcinol (2 TMS) RI 2784, 22.97 min: 5-heptadecenyl-resorcinol (2 TMS) RI 2858, 23.13 min: 5-heptadecyl-resorcinol (2 TMS) RI 2880, 23.67 min: 5-heptadecenyl-methyl-resorcinol (2 TMS) RI 2953, 23.86 min: 5-heptadecyl-methyl-resorcinol (2 TMS) RI 2979. Abbreviations: *Cg*, *Corynebacterium glutamicum*, *Gm*, *Gimesia maris*, *Sf*, *Saltatorellus ferox*; RI, retention index; TMS, trimethylsilyl resdiues.

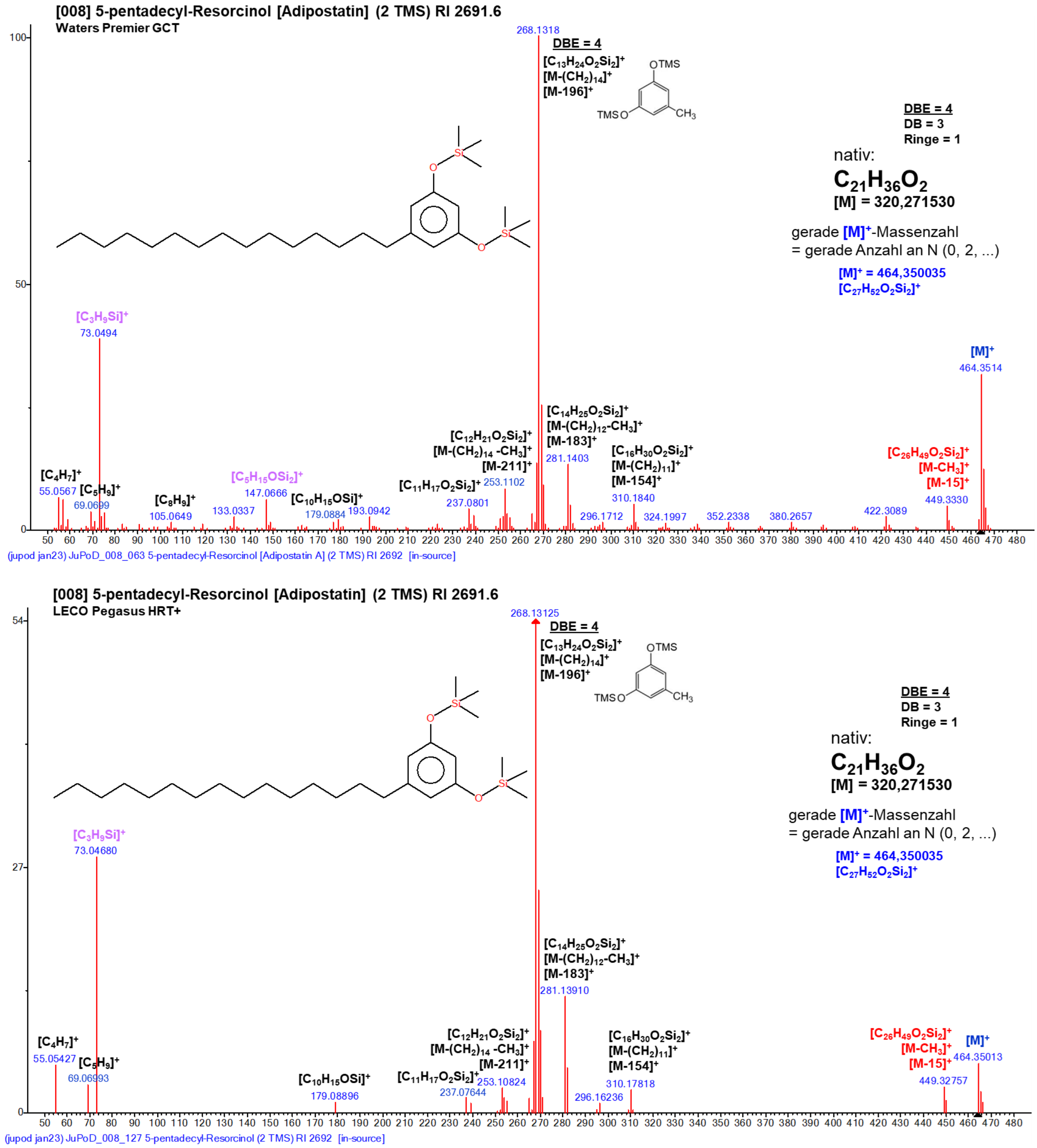

Figure S3. EI+ spectra of derivatized 5-pentadecylresorcinol (2 TMS). The spectra obtained with the Waters GCT Premier mass spectrometer (top panel) and with the LECO Pegasus HRT+ mass spectrometer (bottom panel) are shown. Abbreviations: DB, double bonds; DBE, double bond equivalents; RI: retention index, TMS: trimethylsilyl residue.

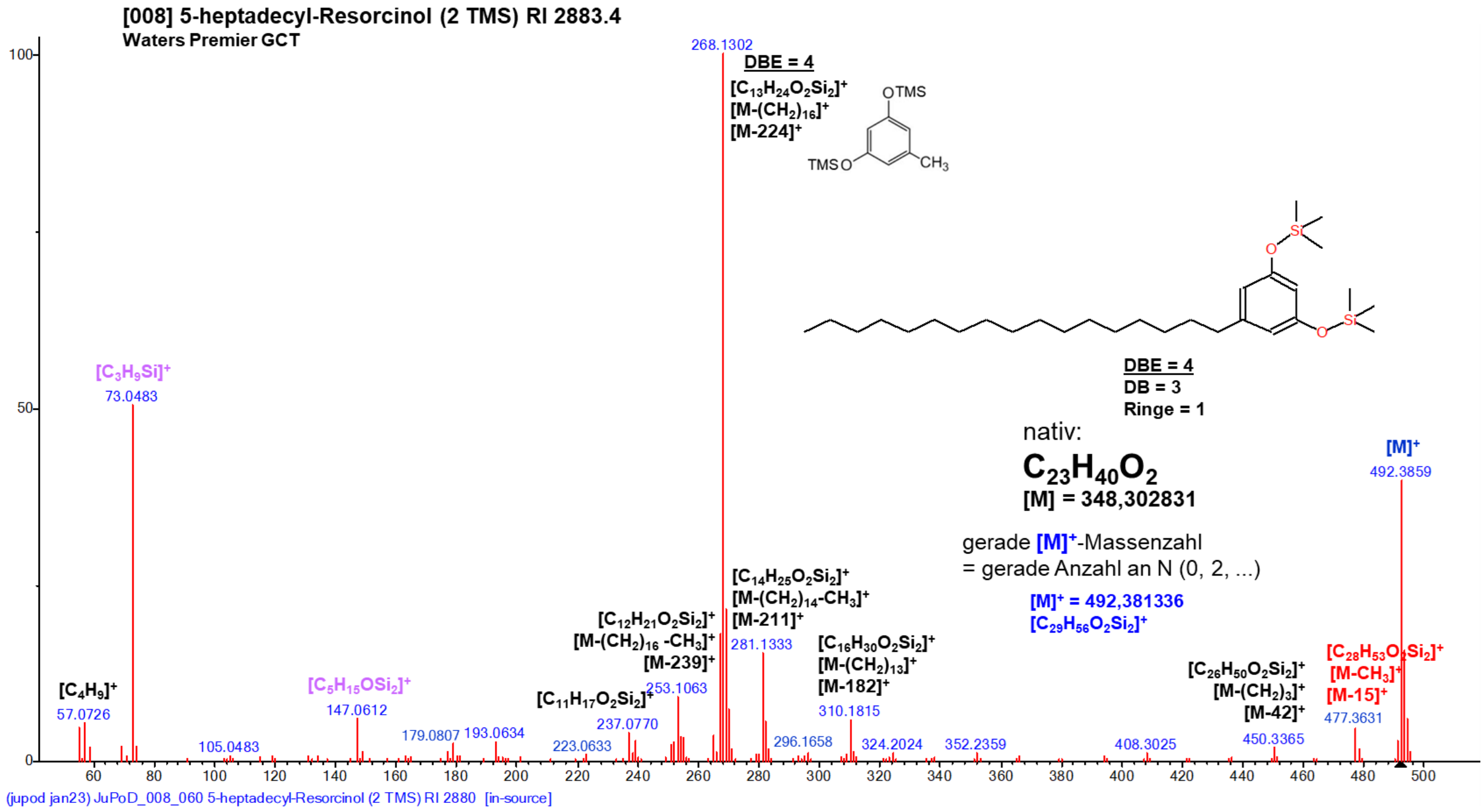

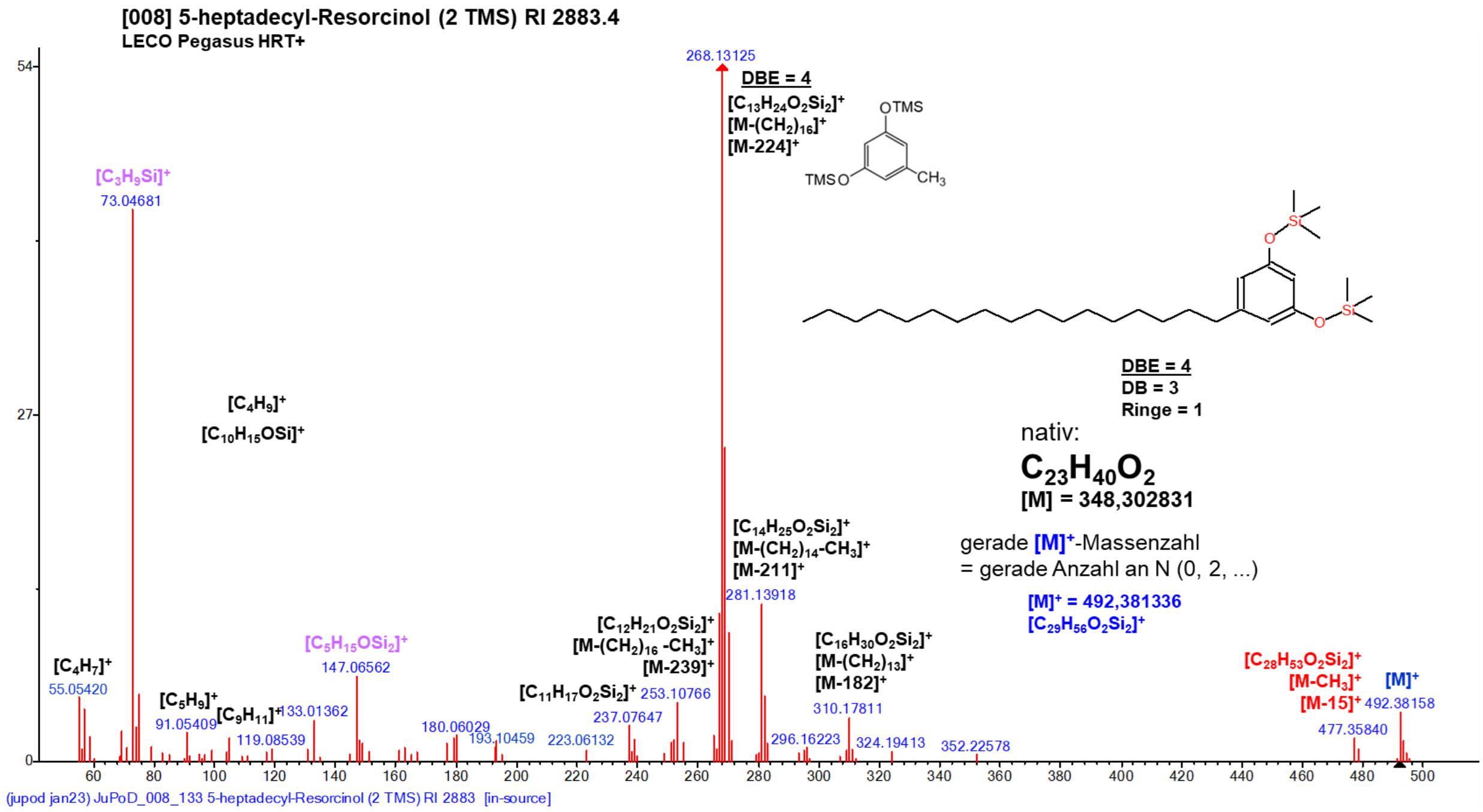

Figure S4. EI$^+$ spectra of derivatized 5-heptadecylresorcinol (2 TMS). The spectra obtained with the Waters GCT Premier mass spectrometer (top panel) and with the LECO Pegasus HRT+ mass spectrometer (bottom panel) are shown. Abbreviations: DB, double bonds; DBE, double bond equivalents; RI: retention index, TMS: trimethylsilyl residue.

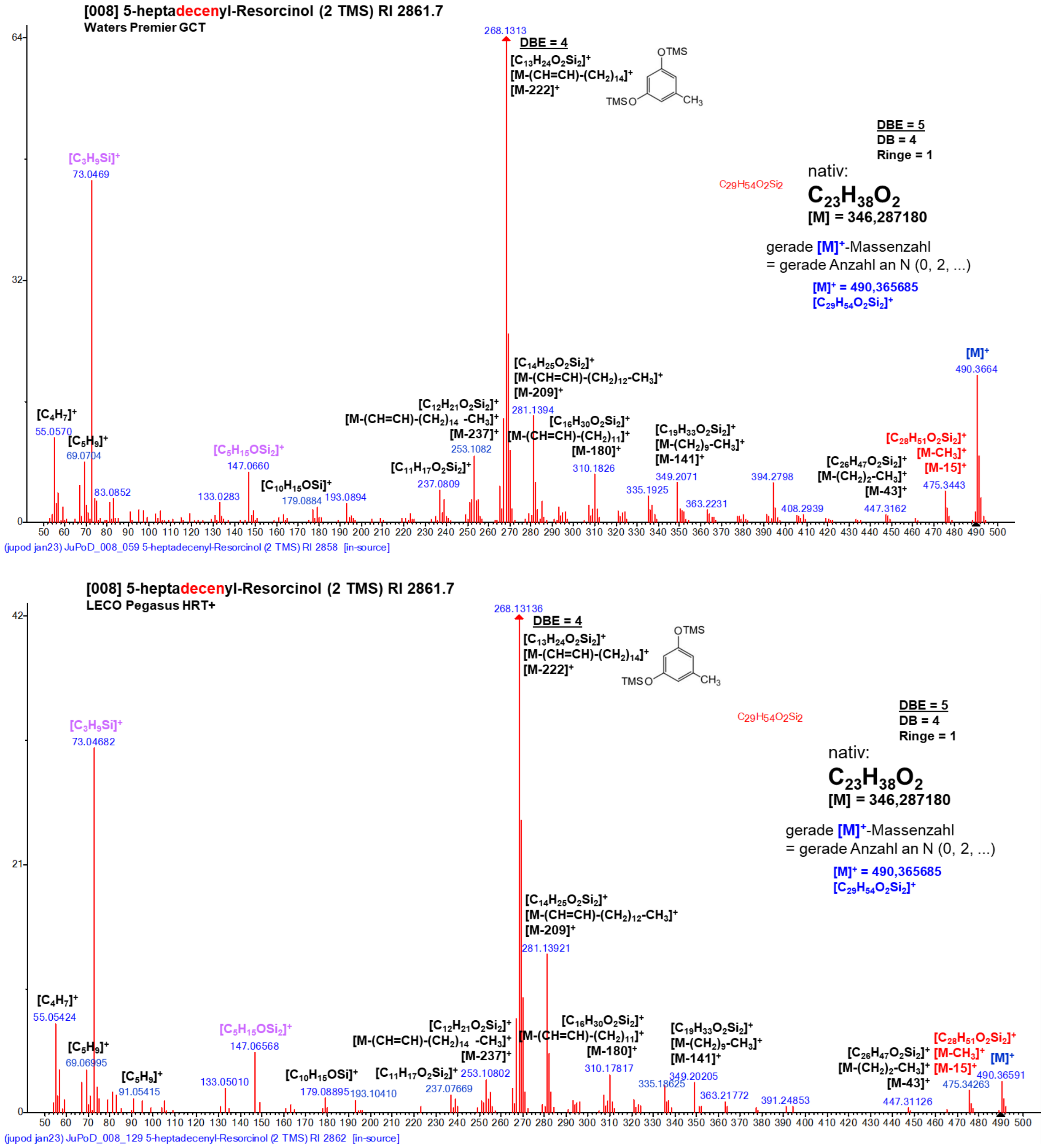

Figure S5. EI$^+$ spectra of derivatized 5-heptadecenylresorcinol (2 TMS). The spectra obtained with the Waters GCT Premier mass spectrometer (top panel) and with the LECO Pegasus HRT+ mass spectrometer (bottom panel) are shown. Abbreviations: DB, double bonds; DBE, double bond equivalents; RI: retention index, TMS: trimethylsilyl residue.

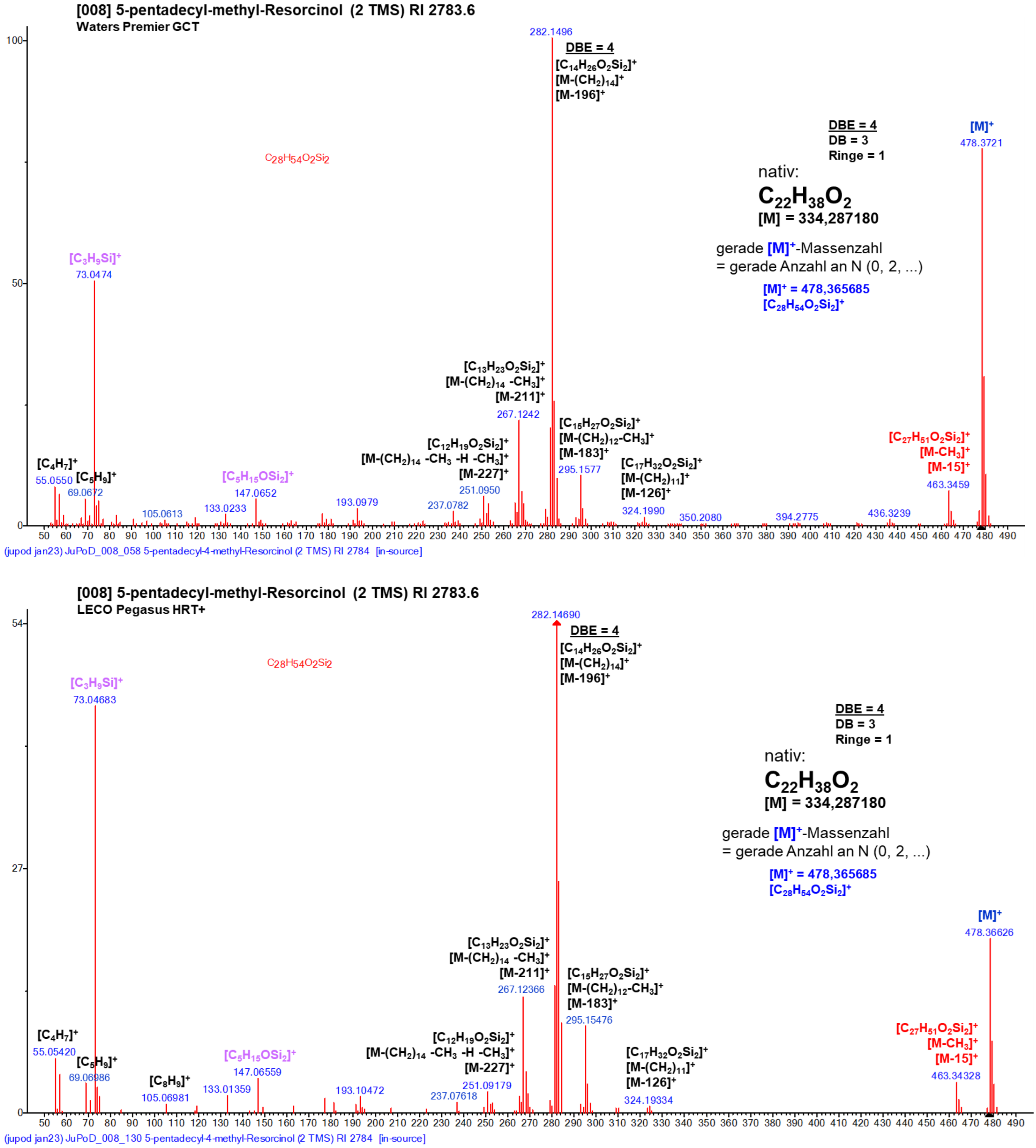

Figure S6. EI$^+$ spectra of derivatized 5-pentadecyl-methylresorcinol (2 TMS). The spectra obtained with the Waters GCT Premier mass spectrometer (top panel) and with the LECO Pegasus HRT+ mass spectrometer (bottom panel) are shown. Abbreviations: DB, double bonds; DBE, double bond equivalents; RI: retention index, TMS: trimethylsilyl residue.

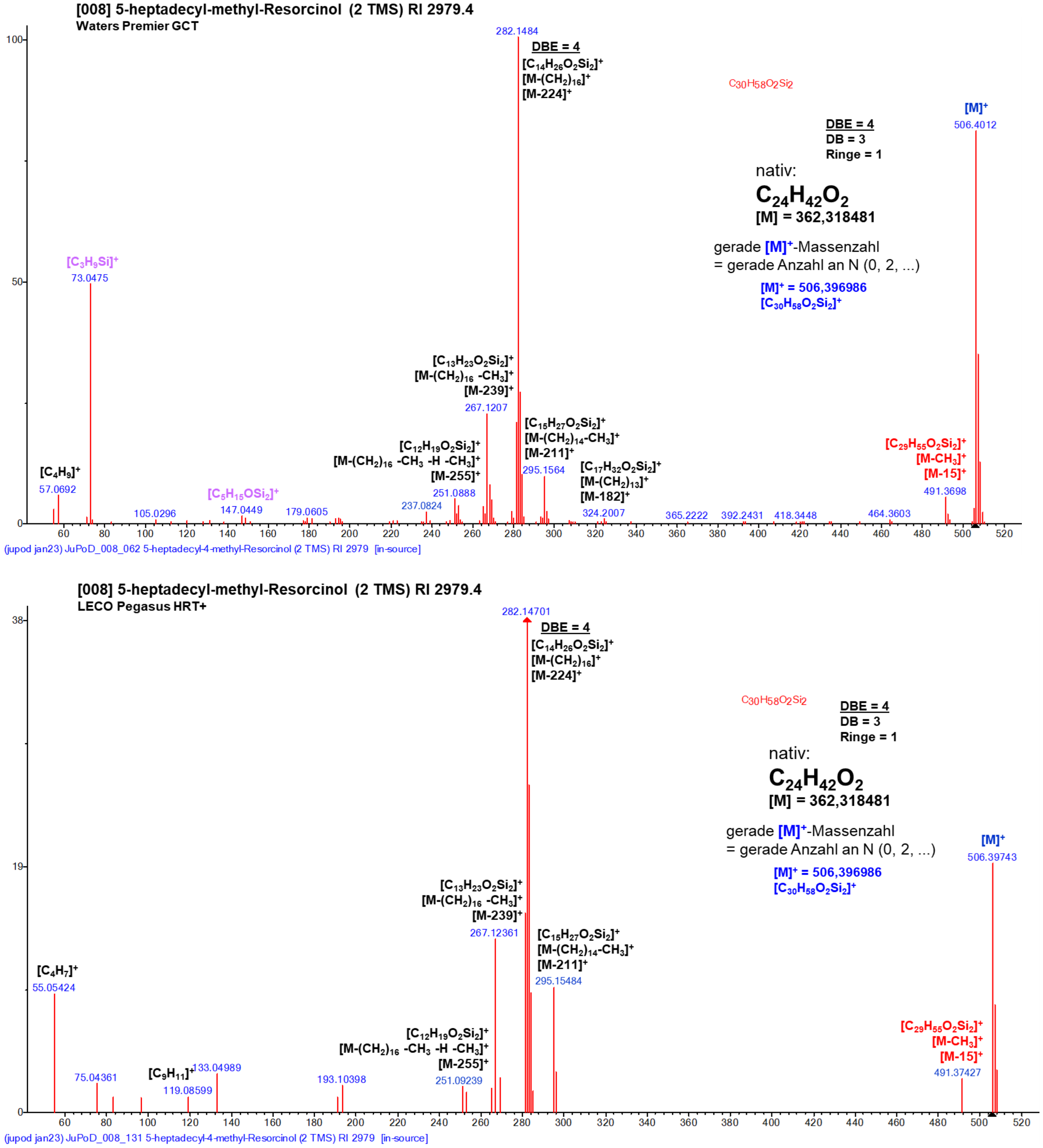

Figure S7. EI$^+$ spectra of derivatized 5-heptadecyl-methylresorcinol (2 TMS). The spectra obtained with the Waters GCT Premier mass spectrometer (top panel) and with the LECO Pegasus HRT+ mass spectrometer (bottom panel) are shown. Abbreviations: DB, double bonds; DBE, double bond equivalents; RI: retention index, TMS: trimethylsilyl residue.

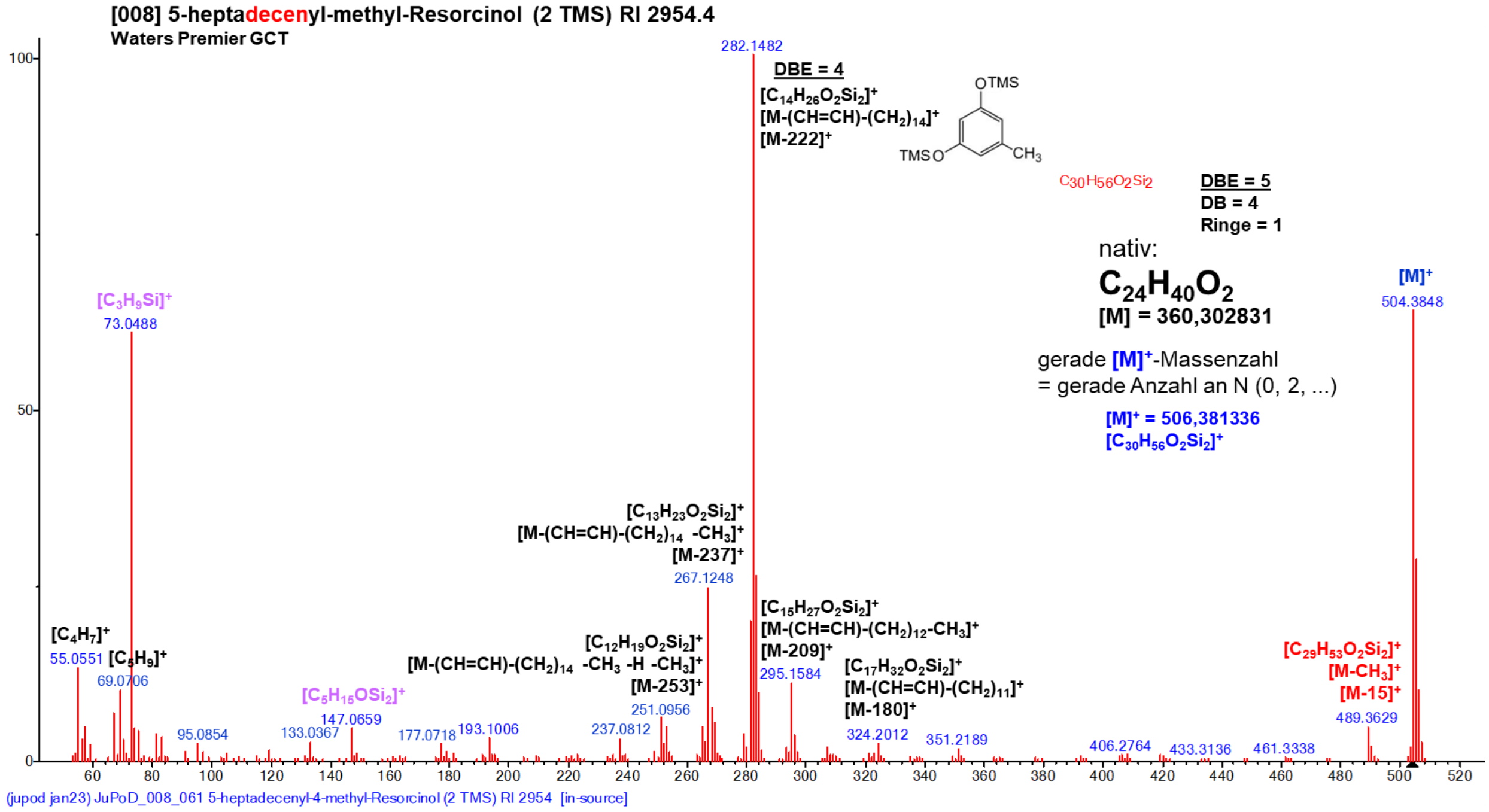

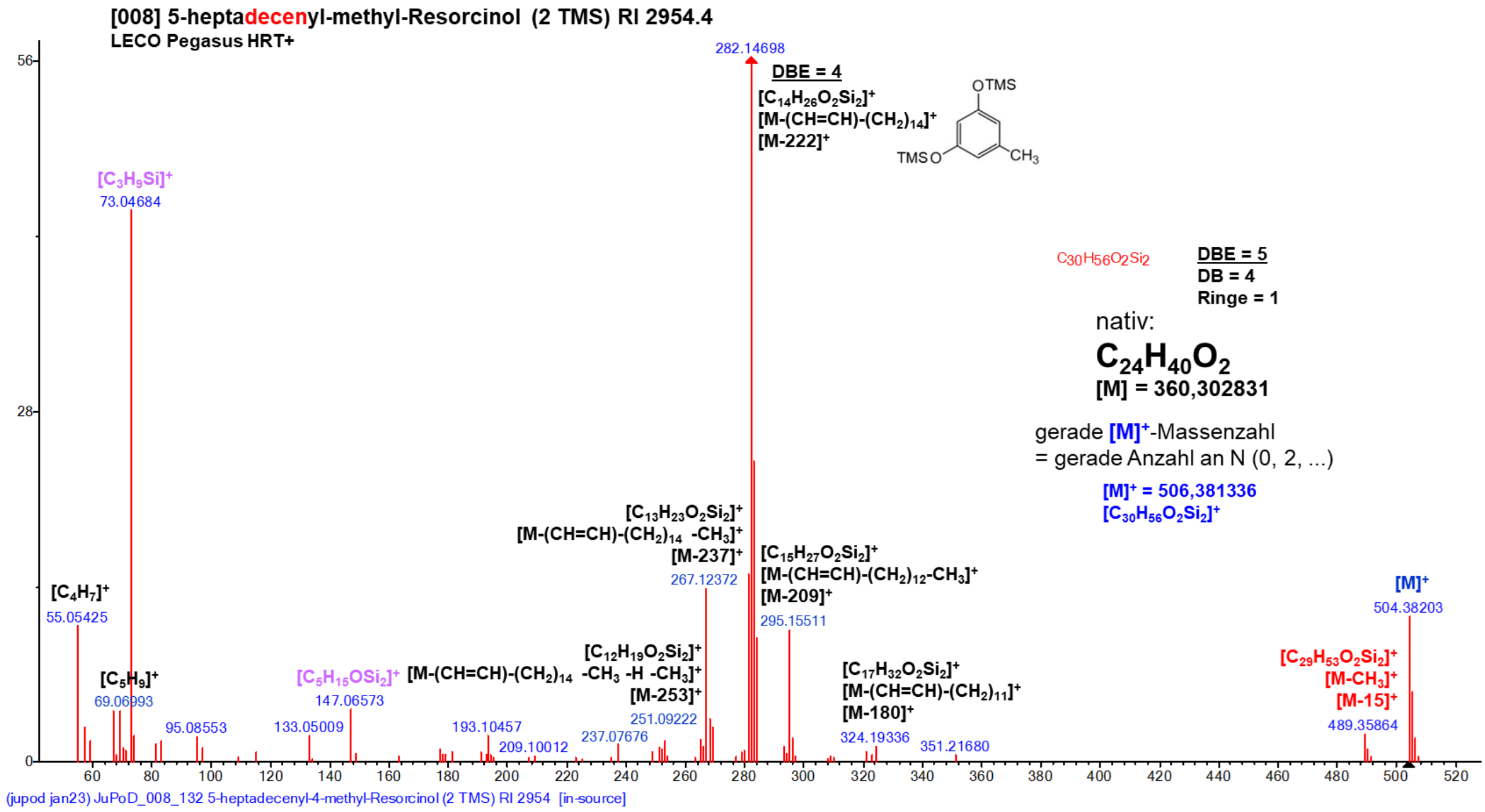

Figure S8. EI+ spectra of derivatized 5-heptadecenyl-methylresorcinol (2 TMS). The spectra obtained with the Waters GCT Premier mass spectrometer (top panel) and with the LECO Pegasus HRT+ mass spectrometer (bottom panel) are shown. Abbreviations: DB, double bonds; DBE, double bond equivalents; RI: retention index, TMS: trimethylsilyl residue.

***S. maiorica* ΔMal15_23020::cat** **_S. maiorica_ ΔMal15_51000::cat**

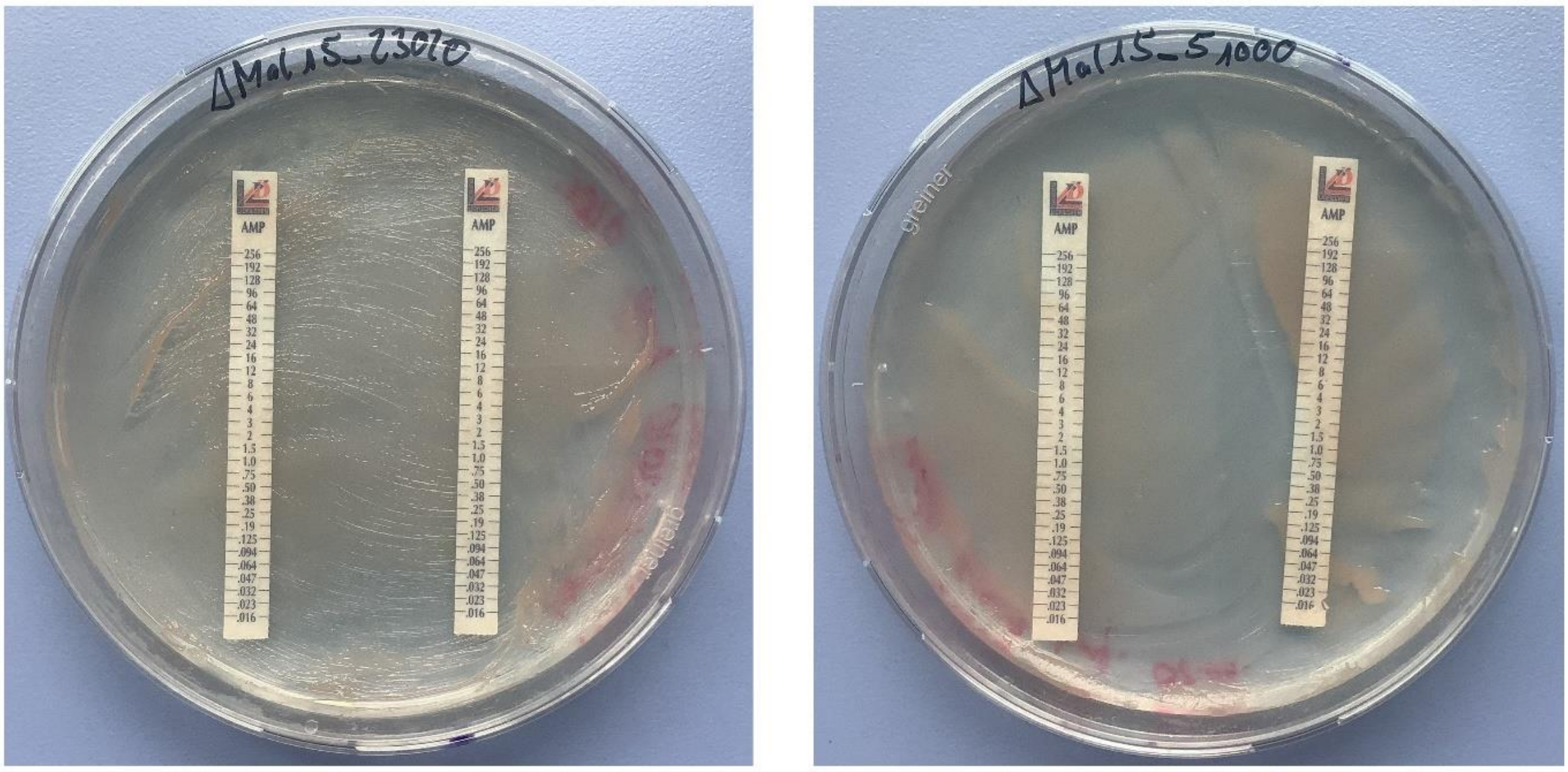

Figure S9. Plate assay for the determination of minimal inhibitory concentrations for ampicillin with constructed *S. maiorica* gene deletion mutants. The two constructed strains were streaked on M3H NAG ASW plates with 34 mg/L chloramphenicol. The plates were incubated with MIC stripes containing ampicillin (0.016-256 mg/L) for two days at 28 °C. Abbreviations: cat, chloramphenicol acetyltransferase (chloramphenicol resistance gene).

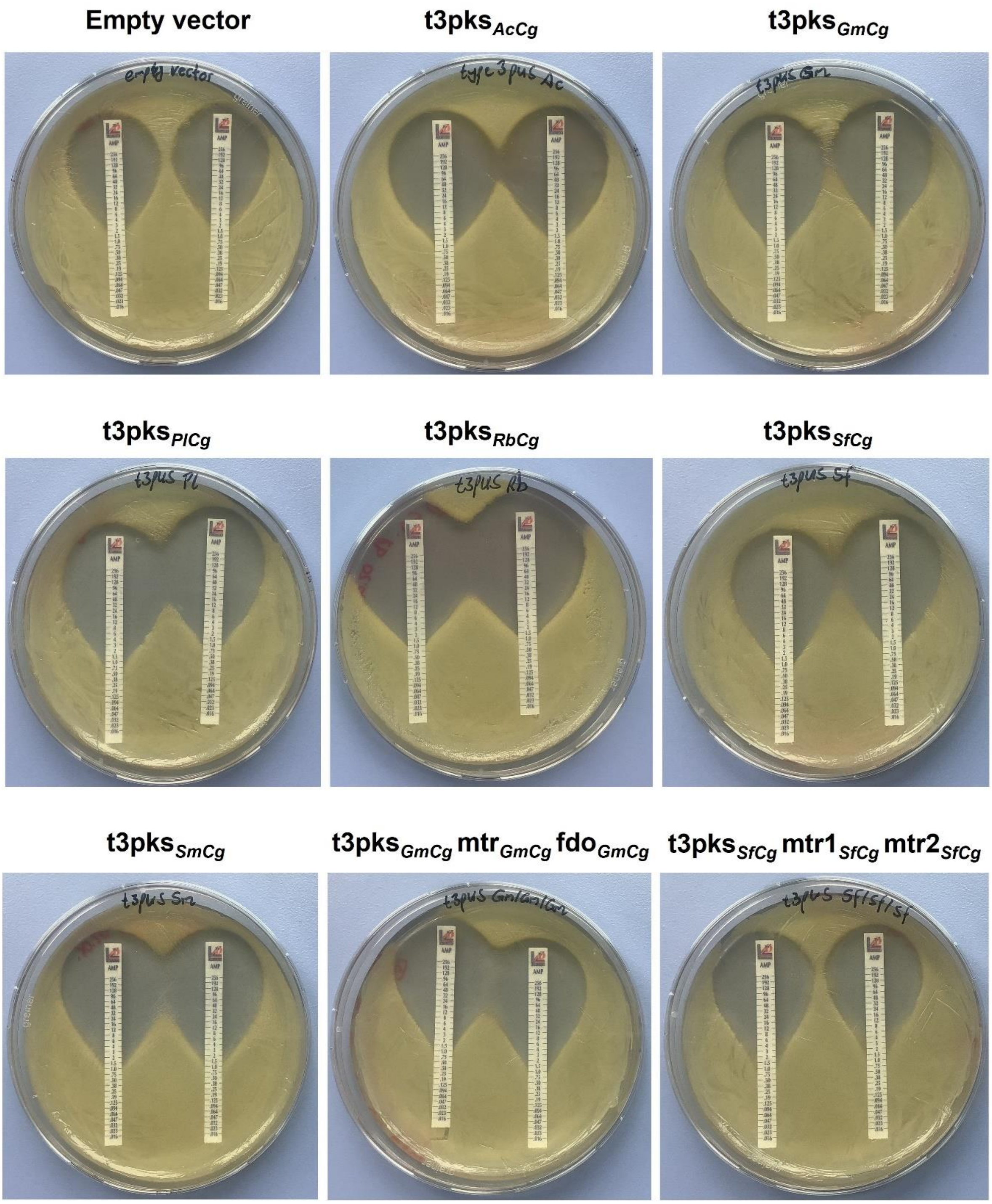

Figure S10. Plate assay for the determination of minimal inhibitory concentrations for ampicillin with constructed *C. glutamicum* strains expressing planctomyete-derived genes. Cell material of the induced *C. glutamicum* cultures (72 h after inoculation) was streaked on CGXII agar with 4% (w/v) glucose, the required antibiotics for plasmid maintenance and 1 mM IPTG. The plates were incubated with MIC stripes containing ampicillin (0.016-256 mg/L) overnight at 28 °C. Abbreviations: *Ac*, *Alienimonas californiensis*; *Cg*, *Corynebacterium glutamicum*, *Gm*, *Gimesia maris*, *Pl*: *Planctopirus limnophila*, *Rb*: *Rhodopirellula baltica*; *Sf*, *Saltatorellus ferox*; *Sm*, *Stieleria maiorica*.

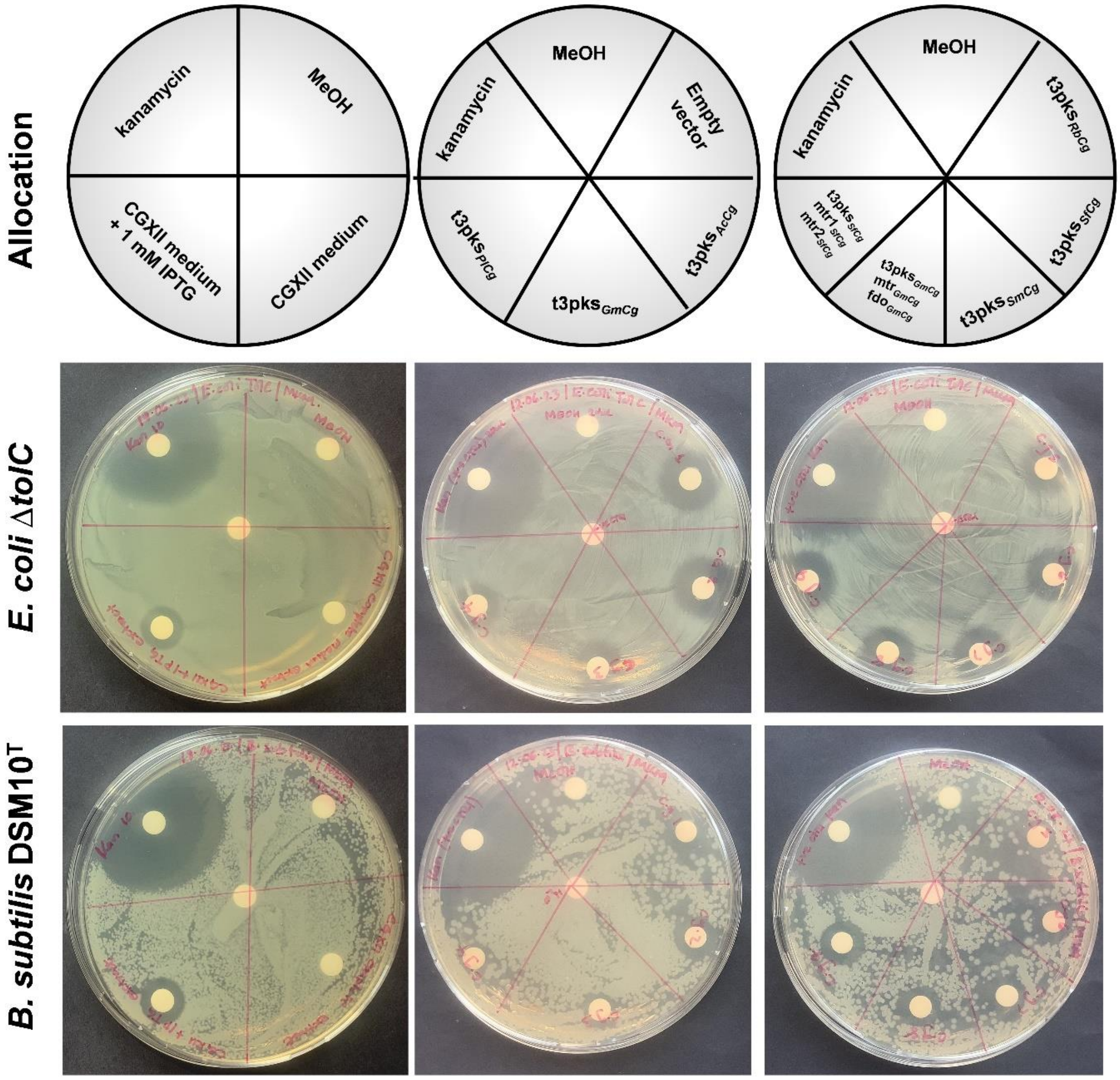

Figure S11. Bioactivity assay with culture extracts of *C. glutamicum* strains expressing planctomycete-derived genes. Fifty mL of the each of the induced *C. glutamicum* cultures (72 h after inoculation) was extracted. The extracts were tested for bioactivity against *E. coli* Δ*tolC* and *B. subtilis*. Kanamycin was used as a positive control. Abbreviations: *Ac*, *Alienimonas californiensis*; *Cg*, *Corynebacterium glutamicum*, *Gm*, *Gimesia maris*, *Pl*: *Planctopirus limnophila*, *Rb*: *Rhodopirellula baltica*; *Sf*, *Saltatorellus ferox*; *Sm*, *Stieleria maiorica*.

Sequences of synthesized genes used in this study. The open reading frames of the codon-optimized genes coding for the type III PKS (t3pks), oxidoreductase (fdo) and methyltransferase (mtr) are provided below. Abbreviations: *Ac*, *Alienimonas californiensis*; *Cg*, *Corynebacterium glutamicum*, *Gm*, *Gimesia maris*, *Pl*: *Planctopirus limnophila*, *Rb*: *Rhodopirellula baltica*; *Sf*, *Saltatorellus ferox*; *Sm*, *Stieleria maiorica*

>t3pks$_{AcCg}$
ATGTCGGGTATTGGAACTGCCTTGCCAGCTGGATGGATCGAACAGGGAGATACTGGTGTA
CTGGCCGGACGGACTGGACCGCCAGCTGGTCCCGACGGAGAAGCGGGCCGTCGCCGTATC
GCAGCCCTCTACCGCAAGGTGGGAGTGAAGAAACGTCACCTGGTTCTTGTGGACCCAGAT
TCGTCTGGTACGGAACCCGATCGCGTCCCATTCTATCCCCCAGGACCGGGTACTCATGCA
GGAGCGCCCACGACTGGTGATCGGATTATCGCGTACGAAAAACACGCCGGCCCTTTGGCA
GTACGCGCCGCAGCTGCCGCACTGGCAGATGCTGGAGTGCATCCAGGCCGTGTAACTCAG
AGCGTTACTGTCTCTTGCACGGGTTTTGCTGCCCCCGGAGTCGATTGTGCATTGATCGAA
GACCTCGGTCTTCCGCGCTCGGTGGGTCGCACCCACGTGGGATTTATGGGATGCCATGCT
GCTCTGAACGGACTTCGTGTGGCCGGTGCTTTGTGTGCTGCGGACCCATCTGCGGCCGTG
CTGCTTACCGCCGTGGAATTGTGCAGCCTGCACCATCAGTACACCCCCGACCCTGTAGCT
GATTCTGGTCAAGTTATTGCCAACGCCCTTTTTGCGGACGGAGCAGCCGCGATGTTGTGC
ACTGGCGCAGACTTCCGGCCTGATCCCCCTACCGCGGGAGTGTCGGACGAACCAAAGACT
GCCACGGCTCCCTACCGCCTGCTCGGAAGCGGATCTGTTGTTTTGGCAGATACGGCCGAC
GCAATGACCTGGCGTGTGGGCGACGCGGGCTTCTTGATGACTCTTGAGCTTGCTGTTCCC
GTAGCAATCCGCGCTCATCTGGCAGGTTGGATGGATGAATGGCTCTCTACTTTCGGTTTG
ACCCGTGCTGAGGTCGGCACTTGGGCAGTGCACCCGGGCGGACCCGCGATTTTGGACGCC
GCAGCGGAAGCACTCGGACTCGGCGAACGTGAACTCGACCCGTCGCGCGCCCTTTTGGCT
GAAGTGGGCAACCTCTCCTCCCCGACTATCCTCTTTCTTTTGGACCGCCTCCGCTCTGCT
CGGGGTCTGGCCGGCGCAAAAGCCTCGGACGGCCCTGTAGTCGCAGTGGGATTTGGACCT
GGACTTACGGTTGAAGCTGCCCTGTTTGGTTAA

>t3pks$_{GmCg}$
ATGAGCTTCGAAATTTTGGGTATCGGCACCACTAACCCCGAACATTCAATCCAACAATCG
GAGGCCGCCGTCCACGCCCAATCACTCTCATGTCACGCAGAAAGCACTGAACAACAGCGT
CGTCTTTTGCCTGTACTGTATCGCCGTGCGGGAGTAAAGACGCGTCATTCCGTAGTCCTC
GAAAATTCCTCTGGCGAAGAAGCCGTTCGGCAAACGTTTTACCAACCAGCGGATAGCCCC
GTGGACCTGGGTCCAGCAACGTCTACGCGTATGCAGGAATATGAGAAACATGCTGCCGAG
CTGGCAATCACTGCTGTACGCGAAGCACTGGATTCGGCTAACGTGAACCCGGCGGAAGTG
ACTCAACTTGTGACCGTTAGCTGTAGCGGCTTTAGCGCGCCCGGATTCGATCTTCAGGTC
TTGAAACAGCCGGGTTTTTCACCCGACGTGTCGCGGACCCACATCGGCTTCATGGGCTGT
CACGGTGCGCTGAACGGTCTGCGTGTCGCAAAGTCCTTTACGGATAATGACCCCGAGGCC
TGTGTTGTAGTCTGCGCGGTGGAGTTGTGTTCACTGCACCAGCAATACGGATGGTGCCCC
GACAAGATTGTTGCAAATGCACTCTTTGCTGACGGTGCCGCCGCGGTAGTTGGTAAACAG
AGCCAGTCATCATCTTCGGACCACTGGAAGTTGGTTGCCTCAGGATCAACGGTGGTGCCT
GACTCAGAGGAAATGATGAGCTGGCGTATCGGTGATCATGGATTCGAAATGACGCTCTCC
CCCTTGATCCCCGATTTGATTAAATCGCGCTTGCGTCCTTGGCTTGAGAACTGGCTCGCG
GGTCAGGGTACGTCTATTGAGGAGATCCGTTCTTGGGCGATCCATCCCGGTGGCCCCCGC
ATTCTTACGGCTGTCTCTGAAGCTGTGGGATTCGATGAGGAGAAATTGGTCCCTTCTCGG
GCAATTCTTGCTGAGTTTGGTAACATGTCATCTCCAACCGTGCTGTTCATCCTGCAACGC
CTTCAGGCGACTCGCACCGCTCGCCCTTGTGTTATGTTGGGCTTCGGCCCCGGCCTTACT
ATTGAGGCTGCTCTTGTCAAATAA

>t3pks$_{PlCg}$

```
ATGGCAATGCTGATCTCAGGTATCGGTACTGCCGCACCTGCATCCCGTATCCCCCAAATG
GATGCTTACAGCGCTATGTCGAATACTTGCTGTGATTCGGATGAACATCGCCGTGTTATG
GAGATGATTTACCAAGGTTCCGGCGTCAAGCACCGTGGCTCAGTTCTCGTAGGCGCCGAG
CCCGAGGACTTTATTCGTGCTGATGACCGCGACGAGTTCTTTTTTTACGAGCGCGAGAAC
GAAGAGGATTACGGTCCATCGACCCAGGAGCGGATGCGGCAATATGAACAGCACGCACTG
CCATTGGCTATCAAGTCATCCGTCGCGGCACTTCGGGATGCCGGACAGGACGCACGGGAG
ATTACTCATATCGTCACGGTTTCGTGCTCAGGCTTCAATAGCCCCGGCGTCGATATGGGA
CTGATTGAAGAACTGGGCCTTAACCGGAATACTTCACGCACTCATGTTGGCTTCATGGGT
TGCCACGGCTCTTTCAACGGATTGCGTGTTGCGCACGGATACACTGCGTCTGACCCCGAT
GCTGTCGTGCTCATGTGCTCTGTCGAACTCTGCTCTCTTCACCATCATTACGGATGGACC
ACCGACAAAGTAATTGCTAATGCACTGTTTGCTGATGGCAGCGGCGCGGTAATTTGCCGG
TCGAGCAAAATCGAAGACCGTAAGCCTGCTTACCAGTTGATTCGGAGCGGTTCCTTTTTG
GTTCCAAACACCAAATTTGCAATGTCCTGGCGCATTGGAAATCACGGATTTGAGATGACG
TTGTCTCAAAAAGTGCCCGCTCTGATTGAGGAAAATTTGGTCTCTTGGCTCACTCCATTT
CTTGCTCGCGAAGGTTTGACTATTAAAGACATCGCGGGTTGGGCGATCCACCCTGGCGGC
CCTCGGATTCTCGACTCTTGTCTTGCCGCTCTCTCTCTCTCCAACAGCCACGTGGCACCT
GCTCGTGAAGTCCTCGAAAAGCACGGTAACATGTCGTCTAGCACCATTTTGTTTGTACTG
GATCGCATGCGTGAGGAACGTATTAATGGTCCAGTCGTGGCCCTTGGATTCGGCCCAGGC
CTGGCTATCGAAGCAATGTTGCTGAAAGCCGAGAACATCTAA
```

>t3pks$_{RbCg}$

```
ATGACCGCTCAGATTCTGTCGATTGCCACTGCACAACCAACGCATAAAGCCGATTTGGCA
TTCTCTACTCATTGCGCGCAGAGCATGTCTTGTGAAGACGAAAGCCAAGCGGTAAAGTTG
GCTAAGCTGTACCGTCGCACCGGTGTTGACACTCGCGGATCGGTACTCGTTGAGAAAGGT
GACGCTGGCGAACTTACTCAATCATTCTACCCGCCGATGCAAGACGGATGCGATCGTGGC
CCCACTATGGCGACGCGTAACGAGCGTTTCGCGGAAGAAGCGCCAGCGCTCGCGTGTCGG
GCAGGCCAGGGTGCTTTGGATGGCTCCGGAATCAGCTCTGACGACGTGACTCACGTTGTA
ACCGTGACCTGCACCGGCTTCACTGCGCCCGGTATCGATGTCCAACTTATCGATAAACTG
GGTCTCCCGATTACGACTCAACGTATCCAAGTCGGCTTCATGGGATGCCACGGACTTGTA
AACGCGCTCCGTACCGCGCGCGGACTTGTAGCCGCTGACAGCGATGCCGTCGTCCTTATT
GTCTGTATCGAGCTTTGTTCCCTCCATTATCAATATGGTTATGACGCCCAGCGTATCGTA
TCTGGCTCCCTGTTCGCGGATGGATCAGCAGGATTGATTGTAGCCGGTGACGATTGTCCC
GCCTCGGGCGTTGATACGCCCCTCGGTGAGATTGTGTCCGTAGGTTCCTGTTTGATTCGG
GATTCTCATGACGCAATGACGTGGCGGATCGGCGACAATGGTTTCATCATGACTTTGGAA
GCGTCGGTGCCCGGTTTTATTGAAACTAATCTTCGTGAGTTTCTCGTCCCTTGGCTCGCC
AAGTCCGGTCTCGATTTGGATTCTATTGGCGGCTGGGCAGTCCATCCTGGCGGAGTGCGT
ATTTTGCAGTCTGTTGAGACCGCCCTGGAGTTGCCGTCGGGCGCTTTGGACGTAAGCCGG
GAAGTCCTCCGCGAACACGGTAACATGTCCTCTGCCACTTTGGGATTCGTTCTGCAAAAA
TTTCAACAGCGGAACGTCCCTGGCCCCTGGTTGATGCTGGGTTTCGGTCCTGGCTTGGAA
ATCGAGGTTGCAGTTGTCCGCTAA
```

>t3pks$_{SfCg}$
ATGCGTATCGCTTCCGTCGGTCGTGCCCTGCCAGAACATGTAGCCTCACAGCAAGAGGTA
ACGGCGGCTTTGATGCAGATTTGGTCTTCGCGGAAATCCGTCACTGACCGTCTGCCGAAG
TTGCTGGAAAACACGCGGGTAGAGCAGCGCCACCTGGTGATGCCGATCGAAAAGTACGGA
GAGATCGGCACGTTCGGTGAATGTAACGATTTCTGGATCGAGTGCGCCAAGGATCTGGGT
GAAAAAGCGATCTTGGAGGGCCTGGAGAAGGTTGGTTTGGGCCCTCAAGATATTGATGCA
ATCTTCACTGTCACGGTAACCGGTCTTGCGTCACCCTCCCTTGATGCCCGCTTGGTAAAC
CGTATGGGACTGCGTGACGATGTGAAGCGTACTCCCATCTTTGGCCTCGGTTGCGTGGCG
GGAGTTGCTGGACTTAGCCGTGCTGCGGACTATGTCAAAGCCTATCCAGATCAGGTAGCT
GTACTGCTCTCTGTCGAGTTGTGCAGCTTGACCTTTCAACCAGGAGACCATTCGGTTGCG
AACTTGATCAGCTCGGGCCTGTTTGGAGACGGAGCTGCCGCGGTGGTTCTCGTGGGAGAG
GAGCGTGCGCGGCGTATGGGTCTTGAGGGCGGTCTCCGTGTGCTCGACACGCGGAGCATT
TTTTACAAGGACACGGAGAACATTATGGGATGGCGGATCTCAGAAGGAGGATTCCAAATT
GTACTTTCACCCGCTGTTCCGGAAGTTGCAAAAGAACGGCTTTCTCCTGGCGTGGACGCG
ATGCTTGAGAAACATGGAATGCAACGGGACGATGTCGCAAGCTGGATTTGTCACCCAGGT
GGACCGAAGGTTCTGTCGGCGATGCAGGAGGGTCTTGGTTTGGACGATTCAAAAGTACAA
CACTCGTGGGACGCACTTGCTCAGCTTGGCAATTTGTCCAGCGCATCTGCACTGATGGTA
TTGCGTGCTCATCTGGATGCGGGCTCACCCGGCCAATTCAAGCGGGGCCTGGCTCTTGCT
ATGGGACCCGGCTTCTGTTCAGAGTTGCTTCTGTTTGAGTGGTGCTAA

>t3pks$_{SmCg}$
ATGATCATCGACGGTTTGGGCACTGCAACTCCTACTCACGGAATTTCGCAGTCTAGCGCC
GCACGCATTGCCCAGACGTTGTGTTGTGATAACCCCAAGCAGAGCCAACTCCTCTCCATT
CTCTATCAACGCTCAGGTGTGAATTACCGTCATTCAGTAGCTCTGGAGTCTTCGGAGCCT
CGGTCTGTATCGGACCGTTCCCATCGCGTTGCCGTACAGGCGGCAGGTGGCCGTGTACCG
GCGGCTGCTGATGTTGCGGTGGAAACCCAACCGGTTCGGCAAACTTTCTTTCCACCAGCA
AGCTCACCTGAGGACTGTGGTCCCACCACTGCTCAACGGATGGAGCTTTATGAGCGCCAC
GCCGGACGTCTCGGCGCGCAGTCTGTCGCAAAAGCACTCGACCAGTCCTCCTGTAACGCG
GGCGACATCGACCATTTGATTACGATCTCCTGCACCGGATTCTATGCCCCAGGACTTGAC
GTTTCCATTATTAATCGCTGCGGCTTGAGCCCGTCCGTCGGTCGGACCCATATTGGATTC
ATGGGCTGCCACGGAGCGCTCAACGGCCTCCGGGTGGCACAGTCTCTGTCACAGTCGAAT
CCGCGTCTGCGGGTTTTGCTTTGCGCAGTCGAGCTGTGCACTCTTCACCAACAGTACGGA
TGGCATCCAGAACGTGTCGTGTCGAACGCGCTTTTTGCGGATGGTGCGGCAGCTCTTGTA
GCACGCACCTCGTCAAGCCCCCCGTCAGGACCTAAGGTGATCGCGTCAGGTTCAACCATC
TTGCCCGATAGCGAGGATCTCATGCGGTGGCGCATCGGAGATCATGGTTTTGAAATGACG
CTTAGCCCACGCGTCCCGACTGTAATCCGGCAGAGCCTGCAACCTTGGTTGCGGGATTGG
CTGGCCAAACATCATTTGGCTATCGAGGACATCCGTGGCTGGGCTATCCATCCAGGAGGC
CCCAAAATTCTGCAAGCCTGCGGCGACGCATTGCAATTTCAGCGGGATGAGCTGAGCATT
TCCCGTTCTATTCTTGCGGAATATGGCAATATGTCATCGCCAACTGTCCTTTTTATCCTG
CAACGCCTGCTCCAAGAACCTGACCGTCTCCCTTGTGTTCTCTTGGGTTTCGGACCTGGT
CTCTCTATTGAAGCCGCATTGATTGGATAA

>mtr$_{GmCg}$
ATGAATTTCAAAATCCGTCAGCGTGAGCCAGAATTGATGGATCAACCTGGCCTTTCCGCA
GGCGAGCATGGACGTGCTCTGGCCGGACTGGGCCGCATTAACTGGTGGAGCCGCTCCGAT
GCCATTGTCTGGCCTGCTGTGTTGGAATCGGCACGCCGGCGTGCGGGAAAGCCTTTGCAA
ATCCTCGATATTGCGTCTGGCGGCGGAGATGTAGCACTTTCAATCGCTGCTCGCGCTGAA
CGTGCTGGCATTCCCGTGGAAATCGATGGTTGTGATATCAGCCCTTTCGCTATTAGCTAC
GCTACCCAGCAGGCAGCAGCTCGCGGCCTCGAACAGGTACGGTTCTTTGAATGTGATGTA
CTGACTCAATCCCTCGACAAACAGTACGATGTGGTAATGTGCTCACTCTTCTTGCATCAT
CTTGATGAGGAACCAGCCCAGCAGCTGCTCAAGATTATGGCCGAATCGTCTCGCGAACTG
ATCCTCGTAAATGATCTTTGCCGTTCCCGCGCCGGTTATTGCCTGGCATGGACGGCGTGT
CGTCTCTTGACGCGGTCTCCTGTAGTGCATGCGGATGGACCGCTCTCTGTCGCTGGTGCT
TTCACTGTCAACGAGATTACCGAACTTGCACGGCAGGCAGGCCTTTCGGGTTTTCAGGTG
ACCCGTCACTGGCCACAGCGTTGGTTGTTGAAATGGAGCCGTAAATAA

>fdo$_{GmCg}$
ATGACTTTGACGTCTTCACTGAATCGTGAGACTGCATGTGGTCAGTCTTGGGATGTTATC
GTCATCGGAGCGGGCCCGGCTGGAGCCGTAGCCGCCCGGCAGCTTGCGCTGCAAAAGCTC
CGCACCTTGTTGATTGAGCGTAAAACTTTCCCGCGTTATAAGGTTTGCGGTTGCTGTCTT
AACCAACGGGCGATCAATGCTTTGCAACAGATCGGCTTGGGCCAGCTGCTGGAATCGGCT
GGCGCCGTGCCTCTGACGCAATTCCGTATGCAGTACCAGGGACGCAAAGCAGCAGTTTCG
CTTCCCGGAGGCCTCGCCATTTCTCGTGAGGTATTCGATACCCTTCTTGTCCAAAGCGCA
ATCGACGCTGGTGCGGCGTATCTCCAGGAAACCTCCGCTACCATTGTGGACTCGGCAGAG
AAGCGTTCGTCGCGTACCGTGGAACTGAATTTCCAAGGTCATACGGCCGGTAGCGTCAGC
GCCAAAATCGTGCTCGCGGCGGACGGCCTCGGTCATCCTTCGTTGCAGAATTGCGCGGGA
TTTGACATGCAAGTAGCTTCAGACTCTCGCATTGGTGTTGGAGCGGCCTTCGTAGTTAAA
AACCAGTCGAGCTATCCCGCAGGTTTGATCCACATGGCCATTCATAAGTCAGGATACGTA
GGCCTGGTTCGCGTTGAAGGAGACCTGCTGAACCTGGCGGCAGCTATTGACCGTGACTTC
ATGAAGCAATGTGCTGGTCCCGCAGACGCGGTACTTCAAATTCTTCGCGCTTCAGAGTCA
CCCCTCCTCCCGGAAATGACTGAATCCAACTTCAAGGGCACCCTTCCCGTCACGCGGAAG
ACCTTGCGGCCAGTCGGTAACCGCCTTTTCATCCTCGGTGACGCAGCGGGATACCTTGAG
CCTTTCACCGGAGAGGGAATGGCTAATGCCATTACTGAGGGAATGGCAGTAGCACAGTTG
GTCCCACAAGGACTGCGGCATTGGGATTCAGCACTCGAACAGAAATGGTTGAAAGCCCAT
TACGCGCTGACGGAGGGTCGTCTTTTCTGGTGCCGGTGGCTTGCACGCCTTCTCCGGTCG
CCAGCAGCTGTGGGTTGGGGTCTCCGCTTGTTCCATCTTTTTCCCGGTATGGCACGCCCT
ATCGTAGCACGGCTTAACCATTAA

>mtr1$_{SfCg}$
ATGGCTCAAGCACAGAATGACTTGGAGATTTACGATCGTGAGGCGGATGCCTGGTGGGAT
GGTTCTGATCGTTTTTTCCGGTCCCTTCGTAGCGTTAAAGAGTTCCATTTGGAGCTGCTG
TTGGAACGCTGGGCGAGCGAGCCATCGGGCGCACGTGTAGTTGATCTCGGATGCGGCGGA
GGTTTGTTCTCGATGGCTCTCGCGGATCGGGGTGCCGATGTAGTTGGTGTGGACCTTAGC
GGTCCCTCCCTCGGTTCCGCGGCACGTGAGGCGGGACGGCGCGGTCATGCAGCTCAATTT
GTGCGGGGTGACTTGACTTCGTCGCCAGTACGTGATGGTTGGGCTGACCTTGTTCTCTTG
TCAGATGTCCTTGAACATGTGGAAGATCCGAAGGCCGCTGTGATCGAGGCTGGCCGTCTG
CTGCGGCCCGGTGGCACGCTCTTGTTGAATACCTTCGATCGTCGGCCGGGTGCGGGACTC
CTCGTTGTTACTGTCGCCGAGGGTCTCGGTCTCGTCCCAAAGGGAACGCACGACCCTGCA
ATGTTCGTCCGTCCTGCTGAGGTTGATGACGCAGCTCGTGAAGCCGGTATGACTCGCACT
CACTTGGTTTGGGAGGCGCCATCATTGGTGAAGACGGTCCGGACCTGGACCATCCACATG
CGCCGCGCACCGCGCGGATTCGCGTATACCGCGTTGTATGAGAAGGCACAGGCATAA

>mtr2$_{SfCg}$
ATGACGGAGATTGCCCTTCGCCAACGCGGCCCACGTGATACTGCGCTTGATCCTTCATTG
TCCACCTTGGCACGCCGTGTGATCGCCGGCACGTACGGCTTGGTCAATCACGGCTCCTTC
GTCGTAGCTGTCGCCGCTATGGCATGGGGCCTTTACCACGGTATGTCGCAGGAGGGAGTG
CCGCACCTGCAAGTCGGACTCGAAGGAGGATGGCGCTGGCTTTGGAACGCCCTTCTTCTT
TTGCAATTTCCTCTCATTCATAGCTGGCTTTTGACGGCTCGGGGTCGCCGTTTCATGGCC
CGGCTCGCACCTTTCGGCACCGGACGCACGCTGGGTGCGACCATCTATACTACGTTGGCG
TCCTGGCAGATCTTGTTTGCGTTCTCCGCTTGGGTTCCGATCGGTAAGGCCTCTTGGTCC
CCTGAGGGTTGGCTCCTCCATGCTTGGCAAGTTTTGTTCGCAGGTTGTTGGTTGTTTCTC
GTTAAAGCGCTTAAGGACGGAGGAATGACCCTCCAGACTGGATCTCTGGGTTGGATGGCC
TTGTGGAAAGGCGAGCGGCCACGTTTTCCGGGCTTGCGTACGCAGGGCACCTTTGCGGCG
TGCCGCCAACCTATTTACCTCGCCTTTGCATCTCTTTTGTGGACTGGCCCTGTTTGGACC
GCTGATCATTTGGCGGTGGCCGGTCTCTGGACCGTATACTGCCTGGTAGGACCAATTCAC
AAAGAGCGGCGGTTCCAATCCATGTACGGTGATGCCTTTATTACTTATAAGTCGCGTATC
CCCTATTTCGTGCCCCGTCTGAAAGGACGGTCTCGCACGCCCCAGCGGCCGTAA
